# Non-catalytic-Region Mutations Conferring Transition of Class A β-Lactamases Into ESBLs

**DOI:** 10.3389/fmolb.2020.598998

**Published:** 2020-11-27

**Authors:** Thinh-Phat Cao, Hyojeong Yi, Immanuel Dhanasingh, Suparna Ghosh, Jin Myung Choi, Kun Ho Lee, Seol Ryu, Heenam Stanley Kim, Sung Haeng Lee

**Affiliations:** ^1^Department of Cellular and Molecular Medicine, Chosun University School of Medicine, Gwangju, South Korea; ^2^Department of Biomedical Sciences, Gwangju Alzheimer’s Disease and Related Dementia Cohort Research Center, College of Natural Sciences and Public Health and Safety, Chosun University, Gwangju, South Korea; ^3^Division of Biosystems & Biomedical Sciences, College of Health Sciences, Korea University, Seoul, South Korea; ^4^Aging Neuroscience Research Group, Korea Brain Research Institute, Daegu, South Korea; ^5^Department of Chemistry, Chosun University, Gwangju, South Korea

**Keywords:** extended-spectrum β-lactamase, non-catalytic-region ESBL, ceftazidime, antibiotic resistance, X-ray crystallography

## Abstract

Despite class A ESBLs carrying substitutions outside catalytic regions, such as Cys69Tyr or Asn136Asp, have emerged as new clinical threats, the molecular mechanisms underlying their acquired antibiotics-hydrolytic activity remains unclear. We discovered that this non-catalytic-region (NCR) mutations induce significant dislocation of β3-β4 strands, conformational changes in critical residues associated with ligand binding to the lid domain, dynamic fluctuation of Ω-loop and β3-β4 elements. Such structural changes increase catalytic regions’ flexibility, enlarge active site, and thereby accommodate third-generation cephalosporin antibiotics, ceftazidime (CAZ). Notably, the electrostatic property around the oxyanion hole of Cys69Tyr ESBL is significantly changed, resulting in possible additional stabilization of the acyl-enzyme intermediate. Interestingly, the NCR mutations are as effective for antibiotic resistance by altering the structure and dynamics in regions mediating substrate recognition and binding as single amino-acid substitutions in the catalytic region of the canonical ESBLs. We believe that our findings are crucial in developing successful therapeutic strategies against diverse class A ESBLs, including the new NCR-ESBLs.

## Introduction

Extended-spectrum β-lactamases (ESBLs) are a serious threat to human health due to their enhanced hydrolytic activity against third-generation cephalosporins such as ceftazidime (CAZ), a representative first-line drug for bacterial diseases (Dance, 2014). In general, class A β-lactamases, regardless of subfamilies, share the similar overall structural architecture of their catalytic region (Supplementary Figure S1), which can be separated into two major compartments: the conserved catalytic ensemble and the variable recognition ensemble. The conserved catalytic ensemble involving the hydrolysis of substrate consists of the reactive Ser70 which attacks the β-lactam amide bond; two general bases Lys73 and Glu166 which electrostatically activate the nucleophilic Ser70; the typical oxyanion hole formed by N atoms of Ser70 and Thr237 stabilizing the negative transient acyl-intermediate; the catalytic (or hydrolytic) water coordinated to Glu166 and Asn170, which disrupts the acyl-bond (Page, 2008; Drawz and Bonomo, 2010). On the other hand, the recognition ensemble, consisting of three critical segments including Ω-loop (160–180 in the Ambler system) (Sirot et al., 1997; Celenza et al., 2008; Dobson et al., 2012; Levitt et al., 2012), lid (92–118) (Doucet et al., 2004; Bethel et al., 2006), and strands β3-β4 (230–251) (Du Bois et al., 1995; Giakkoupi et al., 2001; Shimizu-Ibuka et al., 2011; Ruggiero et al., 2017), is mostly responsible for the adaptability of class A β-lactamases toward different types of antibiotics (Supplementary Figure S1). Upon the innovation of drugs, the residues in this ensemble are further varied to accommodate novel substrates, by which canonical catalytic-region ESBLs are to be produced. These three segments surrounding reactive Ser70 in the active site are known to mediate substrate recognition and hydrolysis directly, and these ESBLs originate in hot spot mutations by single amino acid substitutions in one of the segments of the wild-type β-lactamases^1^. The mutations extend the substrate specificity of the enzyme by increasing the flexibility of the Ω-loop (Wang et al., 2002b), the charged state of the lid (Petit et al., 1995), and the interaction of the β3-β4 strands with substrates (Huletsky et al., 1993; Palzkill, 2018), indicating that the functionality and substrate specificity of the class A β-lactamases largely depend on the subtle changes in the size of the binding cleft.

**FIGURE 1:**
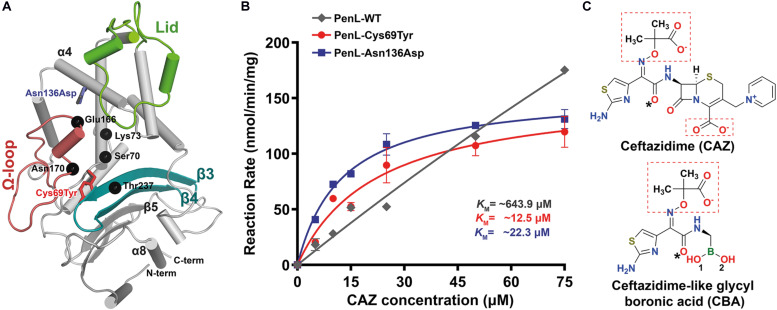
NCR-ESBL-associated mutations and their kinetic analysis against CAZ. **(A)** The overall structure of PenL-WT containing the positions of the NCR-ESBL related mutations (Cys69Tyr and Asn136Asp) with three essential segments (lid, Ω-loop, and β3-β4) and CAZ hydrolysis-associated residues (black dots). **(B)** Kinetics of CAZ hydrolysis. The rate of CAZ hydrolysis by the two PenL-NCR-ESBLs plateaued, indicating the lower *K*_*M*_ value, increased catalytic efficiency *K*_*cat*_/*K*_*M*_ (increased by 3–5-fold, respectively) and enhanced CAZ affinity. Data are averages of triplicate determination in three or four independent experiments from different preparations and presented with standard deviation. The lines are fits of the Michaelis-Menten equation to the data. **(C)** Chemical structure of ceftazidime (CAZ) and ceftazidime-like glycyl boronic acid (CBA). Negatively charged groups harboring oxyimino moiety are marked with red boxes, and asterisks indicate the acetamido hydroxyl groups (see Supplementary Movie S1).

Compared with these canonical ESBLs, the non-catalytic-region ESBLs (NCR-ESBLs) carry single amino-acid substitutions outside of the catalytic segments and potentially hydrolyze CAZ (Gniadkowski, 2001), while conserving residues for substrate binding (recognition ensemble) and hydrolysis (catalytic ensemble) virtually intact (Perez et al., 2007). To date, only two NCR mutations have been identified at the residue 69 (Chaibi et al., 1998; Sam et al., 2009; Dobson et al., 2012) in several class A β-lactamases, including PenL and at 136 (according to the Ambler system) in PenL alone (Dobson et al., 2012), which seem unlikely to involve substrate binding and hydrolysis (Figure 1A). The ESBLs carrying mutations on the position of 69 have been reported from several class A β-lactamases including PenI of *Burkholderia pseudomallei* (Sam et al., 2009), SHV (Giakkoupi et al., 1998; Helfand et al., 2002; Monica A. Totir et al., 2006) and TEM (Samy O. Meroueh et al., 2002; Wang et al., 2002b), wherein the substitutions of Cys69 into Tyr or Met69 into Val, Ile, Leu, Tyr, Phe, or Lys conferred resistance to large size CAZ. Although several structural and kinetics studies suggested that such substitutions on Met69 may disrupt, or perturb the oxyanion hole of the active site (Helfand et al., 2002; Wang et al., 2002b; Monica A. Totir et al., 2006), those studies were unable to address the effect of substitution from Cys69 into bulky Tyr or Phe in case of Pen-type β-lactamase in a molecular level. Another NCR-ESBL carrying a mutation on the position of 136(Asn136Asp) has been reported only in PenL, which displays higher MIC values of CAZ than PenL-WT for *B. thailandensis* (Dobson et al., 2012). PenL (previously called, PenA) from *B. thailandensis* is a class A β-lactamase, which has been extensively studied with regard to its transition into an extended-spectrum β-lactamase (ESBL). This enzyme can evolve *via* a simple nucleotide-substitution, deletion, or duplication mutation to an ESBL, which can hydrolyze third-generation cephalosporins, including ceftazidime. Asn136 locating on helix α4 likely stabilizes Ω-loop by forming hydrogen bonds with the backbone of Glu166 (Yi et al., 2016). Note that Glu166 is one of the critical catalytic residues on Ω-loop for substrate binding in many of class A β-lactamases (Drawz and Bonomo, 2010), thereby forming an energetically unfavorable non-proline *cis*-peptide Glu166 and Xaa167 (Thr167 in PenL, Xaa refer any amino acid on the position as Ambler system). Asn136 is thus crucial for the proper functional orientation of Glu166 proven by the loss of function by substitution of Asn to Ala in the position of 136 of TEM-1 (Banerjee et al., 1998). Therefore, the substitution of Asn136 into Asp in PenL likely disrupts the Ω-loop’s stability, which may affect the substrate specificity of the newly emerged NCR-ESBL. The molecular mechanism of resistance against CAZ by 136 NCR mutation, however, has not been investigated.

In this study, we investigated the detailed molecular mechanisms underlying the acquired activity of the two NCR-ESBLs (PenL-Cys69Tyr and PenL-Asn136Asp) from *Burkholderia thailandensis* against CAZ, which do not directly involve with substrate binding and hydrolysis (Chaibi et al., 1998; Giakkoupi et al., 1998; Dobson et al., 2012; Figure 1A). Our results demonstrate that NCR associated mutations consequently induce the subtle rearrangements on three critical catalytic segments(ensemble) of the PenL, including distortion of the strands β3-β4 and alteration of the electrostatic potentials around the canonical oxyanion hole (Cys69Tyr), enhancement of flexibility of the Ω-loop and increased fluctuation of strands β3-β4 (Asn136Asp). As a result, the substrate-binding cleft of the enzymes is enlarged to accommodate large CAZ.

## Materials and Methods

### Expression and Purification

Genes encoding for PenL-Cys69Tyr and PenL-Asn136Asp were isolated as described previously (Dobson et al., 2012) and sub-cloned into pET28a(+) expression vector. *E. coli* BL21(DE3)-competent cell strain was used to overexpress PenL-WT, PenL-Cys69Tyr, and PenL-Asn136Asp. Transformed cells were grown in Luria-Bertani (LB) media supplemented with 100 μg/mL of kanamycin at 37°C until the OD_600_ reached ∼ 0.6 and induced by 0.5 mM of isopropyl-β-D thiogalactopyranoside (IPTG). After an additional 16 h of incubation at 18°C, cells were centrifuged at 5,000 × *g* and 4°C for 20 min. Cell pellets were resuspended in a buffer containing 50 mM Tris-HCl, pH 7.5, 500 mM NaCl and 10 mM imidazole, supplemented with 0.1 mM phenylmethane sulfonyl fluoride (PMSF), 1 mM dithiothreitol (DTT), and DNase I. The resuspension cocktails were disrupted by high-intensity sonication at 4°C, and the insoluble fractions were separated using high-speed centrifugation (20,000 × *g* at 4°C for 30 min). The soluble fractions, containing the desired proteins with N-terminal 6 × His-tag, were loaded onto Ni-agarose columns. Unbound proteins were washed out with excess buffer containing 50 mM Tris-HCl, pH 7.5, 500 mM NaCl, and 20 mM imidazole. PenLs were eluted using the above buffer containing 250 mM imidazole. To remove the N-terminal 6 × His-tag, the eluted fractions were pulled and dialyzed against the buffer containing 20 mM Tris-HCl, 150 mM NaCl, and 2 mM CaCl_2_, pH 7.5, followed by treatment with 10 U of human α-thrombin (HTI, United States) per 1 mg/mL protein. PenLs were further purified *via* size-exclusion chromatography using a HiLoad 16/60 Superdex 200 pg column (GE Healthcare, United States) saturated with buffer (20 mM Tris-HCl, pH 7.5, and 50 mM NaCl).

### Crystallization and Structural Analysis

The purified PenL-Cys69Tyr and PenL-Asn136Asp were concentrated to 10 μg/μl using a 10,000 Da cut-off Vivaspin centrifugal concentrator (Sartorius). Crystallization was carried out using the hanging drop vapor diffusion method by mixing a 1.2 μl protein sample with a 1.2 μl reservoir solution. Initial screenings were set up using commercial crystallization kits obtained from Hampton (USA) and Rigaku (Japan). Crystals of PenL-Cys69Tyr were grown in a solution containing 200 mM sodium acetate trihydrate and 20% (w/v) polyethylene glycol 3,350 at 4°C. By contrast, crystals of PenL-Asn136Asp were grown in a solution consisting of 100 mM sodium acetate at pH 5.0, 200 mM sodium chloride, and 25% (w/v) polyethylene glycol 3,350 at 20°C. Crystals were transferred to the cryo-solutions comprising a growth solution supplemented with 15–20% glycerol for 30 s, followed by flash-freezing *via* immersion in liquid nitrogen.

To determine the complex structure of PenL-Cys69Tyr and PenL-Asn136Asp with ceftazidime-like glycylboronate (CBA), the cryo-solutions were supplemented with 2 mM CBA, followed by soaking crystals overnight at 4°C before flash-freezing. All the crystals were diffracted at a maximum of ∼1.3 Å resolution. X-ray diffraction and data collection were performed at Pohang Light Source (PLS) beamline 5C (Pohang, South Korea) using the ADSC Q315r CCD detector. Collected data were indexed, integrated, and scaled using HKL2000 (HKL Research Inc.). The structure of the two PenL variants was determined *via* molecular replacement using MolRep (Abergel, 2013) and the structure of PenL-WT (PDB code 5GL9) (Yi et al., 2016) as a reference model. The refinement was carried out with Refmac5 (Murshudov et al., 1997) and phenix.refine (Afonine et al., 2012; Echols et al., 2014). The coordinates and restraints of CBA were generated by eLBOW, and manually fitted to m*Fo*-*Fc* map employing Coot (Emsley and Cowtan, 2004). Details of data diffraction and structural refinement are shown in Supplementary Table S1.

### Determination of Kinetic Parameters

Because of its poor spectroscopic property, ceftazidime (CAZ) can only be measured at a maximum of ∼100 μM. Therefore, appropriate amounts of purified enzymes were mixed with various CAZ concentrations ranging from 5 to 100 μM in a reaction buffer comprising 50 mM potassium phosphate at pH 7.0 supplemented with 20 μg/mL of bovine serum albumin. The absorbance at 260 nm was immediately monitored at 25°C using a cuvette holder temperature controller. The initial velocity of PenL-WT was measured during the first 10 s using a standard procedure, and the velocities (*v*) were fitted to the Michaelis-Menten equation (Supplementary Information). The first-order persistence of reaction by PenL-WT prevented the calculation of *K*_*M*_ and *k*_*cat*_ within the experimental setup range; however, the catalytic efficiency can be estimated by reciprocal plotting, in which the slope of the regression curve is *k*_*cat*_/*K*_*M*_. Data fittings were carried out using in-house Python scripts with the power of SciPy (Oliphant, 2007) library for parameter estimation, and Matplotlib (Hunter, 2007) module for data visualization.

### Molecular Dynamics Simulation

PenL-WT, PenL-Cys69Tyr, and PenL-Asn136Asp were subjected to all-atom molecular dynamics (MD) simulations in explicit solvent using Gromacs 5.0.7 suite (Lemkul et al., 2015) and Gromos96-43a1 force field (Ramos et al., 2019). The coordinate of PenL-WT was obtained from the RCSB Protein Data Bank by fetching the ID 5GL9 (Yi et al., 2016). Molecules were solvated using the TIP3P water model and neutralized with a cubic boundary of 0.10 M of NaCl. Particle mesh Ewald method (Abraham and Gready, 2011) was used to determine the electrostatic interaction of systems, with real space and a *van der* Waals distance cut-off of 10 Å. After energy minimization by 500-step in steepest descent calculation, systems were heated to 300 K and simulated for 50 *ns*. The MD trajectory analysis was performed directly in the Gromacs package, and the RMSD and radius of gyration plotted with 100 frame intervals (Supplementary Figure S8).

## Results

### Biochemical Properties of CAZ Hydrolysis by PenL Wild-Type and NCR-ESBLs

To elucidate the mechanism associated with CAZ hydrolysis by the two PenL NCR-ESBLs (PenL-Cys69Tyr and PenL-Asn136Asp), we first determined their kinetic parameters (Table 1). The two PenL NCR-ESBLs and wild-type PenL (PenL-WT) hydrolyzed CAZ over time in which the CAZ decay was linearly related to time for 10 s (Supplementary Figure S2). Figure 1B illustrates the comparative kinetics of CAZ hydrolysis by the two ESBLs and WT of PenL. The kinetics of CAZ hydrolysis were similar between the two ESBLs, with *V*_*max*_ and *K*_*M*_ values of 156.4 nmol/min per mg and 22.3 μM, respectively, for PenL-Cys69Tyr, and 155.8 nmol/min per mg and 12.5 μM, respectively, for PenL-Asn136Asp. The mutant’s *K*_*M*_ values are 30–60 times lower than that of WT (Table 1), indicating that a stable acyl-enzyme complex may be formed after binding in the mutant ESBLs. Although the *k*_*cat*_ values of the two PenL-ESBLs were lower than that of PenL-WT, their catalytic efficiency (*k*_*cat*_/K_*M*_) was enhanced 3–5-fold compared with that of PenL-WT (Table 1, Figure 1B, and see Supplementary Information). Those biochemical results are likely to correspond to the higher MIC values of CAZ than PenL-WT for *B. thailandensis* (Dobson et al., 2012). Overall, the changes in kinetic property, primarily with the decreased *K*_*M*_, in the two PenL ESBL variants indicate that the two single substitutions led to alternative substrate recognition for the enzyme. Interestingly, the single mutations at non-canonical regions are involved neither in substrate binding or catalysis.

**TABLE 1:** Kinetic parameters of ceftazidime hydrolysis.

Variant	*K*_*M*_	*V*_*max*_	*k*_*cat*_	*k*_*cat*_/*K*_*M*_
	(μM)	(nmol/min/mg)	(s^–1^)	(nM^–1^ s^–1^)
PenL-WT*	643.91.07*	1657.0384.63*	2.00.460*	3.0*
PenL-Cys69Tyr	22.30.98	156.42.51	0.20.003	8.4
PenL-Asn136Asp	12.50.54	155.81.67	0.20.002	14.9

Data are means ± SD of triplicate determination in three or four independent experiments. *Estimated from linear regression.

Next, to examine whether the enhanced CAZ affinity and catalytic efficiency were related to changes in the conformation of PenL-Cys69Tyr and PenL-Asn136Asp, Circular dichroism (CD) spectroscopy was used for comparison with that of wild-type (Supplementary Information). The CD spectra of their respective apo-forms (PenL-Cys69Tyr-apo and PenL-Asn136Asp-apo) showed no significant differences compared with the PenL-WT (Supplementary Figure S3A). However, the CD spectra of the two NCR-ESBLs in the presence of a non-hydrolyzable CAZ analog glycylboronate (CBA) slightly deviated from their apo-forms as well as the PenL-WT-apo form. The degrees of the CD spectral change of the mutants appeared similar to that of WT from CAZ presence. Moreover, the deviations in a spectral difference between the two ESBLs seemed even less than those with CBA, which might result from the rapid hydrolysis of CAZ accompanied by the slight conformational changes (Supplementary Figure S3B). These observations suggested that the conformation of the two NCR-ESBLs was similar to that of PenL-WT; however, the NCR-ESBLs may recognize the substrate CAZ (or CBA) by marginally changing their conformation of the side chain of residues in the active site or recognition ensemble.

### Crystal Structures of PenL-Cys69Tyr NCR-ESBLs

**FIGURE 2:**
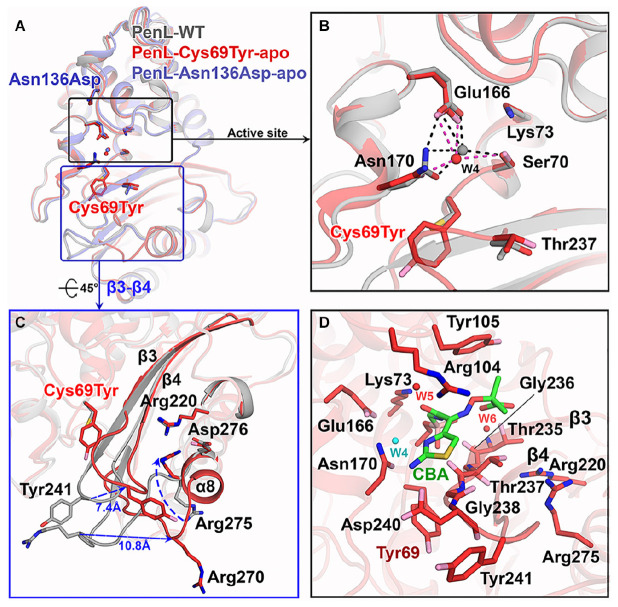
Structure of NCR-ESBLs and Cys69Tyr mutation-induced β3-β4 changes. **(A)** Superposition of structures of the PenL NCR-ESBLs with PenL-WT. The structural similarity over the entire molecule is shown. Typically, the configuration of side chains for catalytic residues is well preserved among the proteins. **(B)** Comparison of active site between WT and Cys69Tyr variant. The catalytic residues from both proteins coordinate the catalytic waters (W4), which coincide in the active site. **(C)** Structural changes in β3-β4 of PenL-Cys69Tyr. Replacement of bulky Tyr at 69 increases the flexibility of τηεβ3-β4 loop (∼7.4 Å at Tyr241) by disassembly of β4. The change induces the dislocation of a loop between β5 and α8 (∼10.8 Å at Arg270) and flip Arg275 on the β5-α8 loop toward the substrate-binding region, resulting in expansion of the active site and altered electronic properties of the oxyanion hole. **(D)** The active site structure of PenL-Cys69Tyr-CBA. Residues on b3 involved in the interaction with CBA, and Tyr105 also move upward for facilitating the entry of large substrate (see also Supplementary Figure S6A). Arg275 keeps its conformation projecting into the oxyanion hole, and Tyr241 moves back to that of WT to some extent.

**FIGURE 3:**
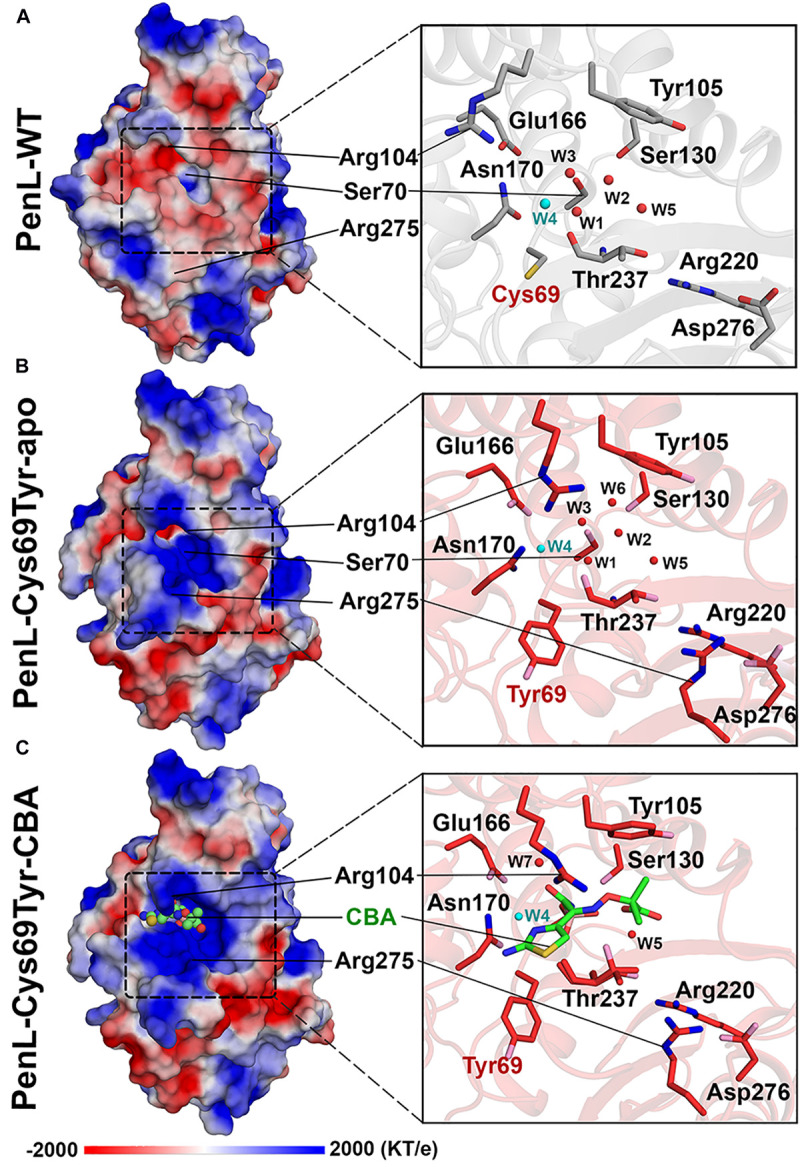
Electrostatic potential changes in the catalytic cavity of PenL-Cys69Tyr. **(A)** Electrostatic property of the active site in PenL-WT. **(B,C)** Electrostatic distribution in the substrate-binding cleft of PenL-Cys69Tyr-apo and CBA-bound form (acylated). Residues such as Tyr69, Se70, Arg104, Glu166, Asn170, Arg275, and W4 catalytic water molecule are actively involved in substrate binding, acylation-deacylation step, and oxyanion hole formation (see Supplementary Movie S2).

To further investigate the conformational changes based on substrate recognition, we delineated four crystal structures of the two PenL NCR-ESBLs: two involving the apo-form and the other two associated with the CBA-bound form (Supplementary Table S1 and Supplementary Figure S4). Consistent with CD spectra, PenL-Cys69Tyr-apo and PenL-Asn136Asp-apo were structurally similar to PenL-WT (PDB ID:5GL9) (Yi et al., 2016) with root-mean-square deviations (RMSDs) of 0.221 and 0.186 Å at their Cα atoms relative to the wild-type, respectively (Figure 2A). In particular, the configurations of the conserved catalytic residues and the catalytic water (W4) associated with hydrolysis coincided with those of PenL-WT (Figures 3B, 2B and Supplementary Figure S5). These similarities suggest that the respective substitution does not structurally alter the active site involved in acylation/diacylation of the β-lactam backbone during hydrolysis (Lamotte-Brasseur et al., 1991; Page, 2008; Brown et al., 2009; Drawz and Bonomo, 2010; Papp-Wallace et al., 2013).

Interestingly, the PenL-Cys69Tyr-apo showed structural variation in the β3-β4 strands compared with PenL-WT-apo (Figure 2C). Notably, the C-terminus of β3 and the N-terminus of β4 in PenL-Cys69Tyr-apo were disrupted in three (Thr237 to Asp240) and two (Thr243 to Gly244) residues, respectively. The disruption in the β-strands was apparently induced by the steric hindrance of bulky Tyr69 against the β3-β4 sheet, resulting in enhanced flexibility and dislocation of the loop by 7.4 Å at Tyr241 (Figure 2C). The increased flexibility of the β4 N-terminus in PenL-Cys69Tyr-apo induced a loss of hydrogen bonds with adjacent C-terminus of β5 and subsequently dislocated the β5-α8 loop away from the corresponding position of Pen-WT-apo by 10.8 Å at Arg270 (Figure 2C and Supplementary Movie S1). Since the β3-β4 segment is one of the recognition ensembles, these changes likely expand the active site of the enzyme to accommodate large substrates such as CAZ. The structure of PenL-Cys69Tyr-CBA shows that the residues ranging from Thr237 to Asp240 of β3 mediated substrate recognition, and the loop interacts with the aminothiazole ring and acetamido backbone, rather than the oxyimino group of CBA (Figure 2D). These results suggest that the NCR mutation induced similar effects as in the known canonical ESBLs. For instance, the dislocation of the loop between β3-β4 strands in TEM-52 ESBL (Gly238Ser on β3) enlarged the active site to bind CAZ without further loop extension and with minimal dislocation (∼2.9 Å) (Papp-Wallace et al., 2012). In addition to structural analysis, we calculated the electrostatic distribution on the surface of proteins (Figure 3). Surprisingly, we found a significant change in electrostatic property from negative to positive in the active site of PenL-Cys69Tyr-apo, which has never been reported in ESBLs. Upon distortion of strands β3-β4, the helix α8 was extended by four residues (Ala272-Arg275) (Figure 2C and Supplementary Movie S1). Accordingly, Arg275 rotated 180° to relocate closer to the Thr237 with a distance of ∼6.7 Å (between N atom of Arg275 and backbone N of Thr237) in the Arg220-Asp276-Thr237 cluster, which is critical for substrate binding and hydrolysis (Papp-Wallace et al., 2010, 2012; Figures 2C,D, 3C). The Thr237 residue in the cluster mainly contributed to the oxyanion hole, stabilizing the tetrahedral intermediate formed *via* nucleophilic attack by catalytic Ser70 (Figures 2C, 3 and Supplementary Figure S5). Note that, based on the established mechanism of β-lactam hydrolysis (Drawz and Bonomo, 2010; Papp-Wallace et al., 2013), the N atom of Ambler residue 237 (i.e., Thr237 in this case) participates in the formation of the oxyanion hole, stabilizing the tetrahedral intermediate of a substrate with class A β-lactamase. Besides the residue 237 in the cluster, Arg220, whose configuration was not changed between PenL-WT and PenL-Cys69Tyr, is directly in close contact with Thr237 (∼4.4 Å) (Figures 2C,D). Remarkably, the Arg275 in the Cys69Tyr mutant positions adjacent to Arg220 within ∼3.6 Å, which may strengthen the positive charge around the oxyanion hole (Supplementary Figure S9C). Consequently, the accumulation of additional basic amino acid (i.e., Arg275) increased the positive electrostatic potential around the oxyanion hole (Figure 3 and Supplementary Movie S2). Notably, due to the presence of two negatively charged groups on CAZ (Figure 1C), the increased net positive charge in the PenL-Cys69Tyr active site may enhance the binding affinity with CAZ, which is consistent with the kinetic data (Table 1). Taken together, the findings indicate that the Cys69Tyr mutation in class A β-lactamase affects the structural interaction between β3–β4 strands and substrate by enlarging the substrate-binding cleft of the enzyme and altering electrostatic property around the oxyanion hole in the active site. These changes in the NCR-ESBL may result in enhanced recognition of large CAZ without affecting the conserved catalytic residues of class A β-lactamases located in the active site for hydrolysis.

### Crystal Structure of PenL-Asn136Asp NCR-ESBL

**FIGURE 4:**
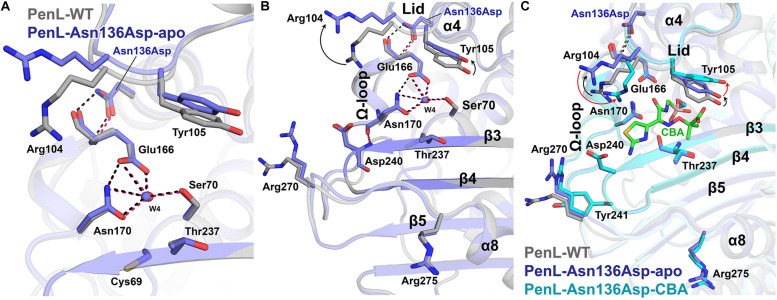
Structure of Asn136Asp NCR-ESBL and changes on lid. **(A)** Superposition of structures of the PenL-Asn136Asp-apo with WT. The side chain configurations of catalytic residues are superimposed regardless of the mutation. **(B)** Conformational change on the lid segment. Asp104 and Tyr105 in apo-form were displaced from substrate-binding cleft compared to those in WT, resulting in the expansion of the active site’s size. **(C)** Structure of PenL-Asn136Asp-CBA. The two residues, including Arg104 and Tyr105, underwent subsequent structural changes upon CBA binding. Moving Arg104 toward CBA (red arrow) and lifting Tyr105 upward further from the active site (red arrow) are likely to provide a large substrate like CAZ with adequate space. However, the β3-β4 and residues that underwent conformational changes in the Cys69Tyr variant remained unchanged (see Supplementary Movie S3 and compare Figure 2).

Similar to Cys69, the residue Asn136 is positioned outside the essential catalytic segments and not involved in the catalysis of antibiotics. However, a novel ESBL variant carrying the substitution of Asn136Asp was found only in PenL (Dobson et al., 2012) and also exhibited an increase in CAZ hydrolysis activity (Table 1 and Figure 1B). Asn136 is located proximally to active site cleft so that its side chain forms a hydrogen bond to the backbone of Glu166 on Ω-loop and stabilizes the energetically unfavorable non-proline *cis*-peptide (Berg et al., 2012) between Glu166-Thr167 (Figure 4A). Notice that Glu166 is a critical catalytic residue that involved in both the activation of the Ser70 and deacylation step in β-lactam hydrolysis (Lamotte-Brasseur et al., 1991; Guillaume et al., 1997; Meroueh et al., 2005; Page, 2008; Drawz and Bonomo, 2010; Papp-Wallace et al., 2013). Asn136 thus appears to be responsible for the proper orientation of Glu166 and Ω-loop. Evidently, the loss of the stabilization by Asn136 (i.e., by mutation Asn136Ala) resulted in the functional deficiency of a class A β-lactamase (Banerjee et al., 1997, 1998). The replacement of Asn136 to aspartate abolishes a hydrogen bond formed with Glu166 in WT (Figure 4A), which probably leads to an increase in Ω-loop flexibility. To our surprise, the structure of PenL-Asn136Asp demonstrates no significant difference in comparison with PenL-WT (Figure 4A). Nonetheless, PenL-Asn136Asp can accommodate the CBA into its active site wherein the conformation does not change substantially (Figure 4C and Supplementary Figure S6). Residue Arg275 also adopts the same configuration as that of the wild-type enzyme, implying no such alteration of electrostatic distribution around active site cleft, as seen in PenL-Cys69Tyr (Figure 4B). However, the large-sized CAZ may bind to the active site with the help of the change in lid segment, although no such considerable distinction between PenL-Asn136Asp and PenL-WT, as well as between PenL-Asn136Asp-apo and PenL-Asn136Asp-CBA, is observed. Two residues, including Arg104 and Tyr105, in the lid of PenL-Asn136Asp-apo, appeared to move away from the active site than WT (Figures 4B,C). In PenL-Asn136Asp-apo, residue Arg104 swung away by ∼4.0 Å from the corresponding position of WT, whereas Tyr105moved upward by ∼1.3 Å. Then, Arg104 returned toward the active site when the CBA bound. Instead, the Tyr105 moved further upward by another 2.0 Å in the CBA bound form (Figure 4, Supplementary Figure S7, and Supplementary Movie S3). These observations indicate that Asn136Asp mutation may affect the conformation of the lid segment to expand the size of the substrate-binding cleft and receive large size CAZ. Interestingly, the changes in the lid resemble that the effects of mutation on Ω-loop propagated into those residues to enlarge and accommodate CAZ (Yi et al., 2016). Therefore, the mutation at 136 may cause the instability of the Ω-loop.

An interesting question can then be raised here on how the relatively negative CAZ can be attracted to an electrostatically negative substrate-binding cleft of the PenL-Asn136Asp (Supplementary Figure S6) like that of PenL-WT. Taken together with the CD spectra data and kinetic analysis, these observations indicate that the binding of CAZ into the binding site might be governed by latent factors other than the electrostatic attraction, which was not be revealed in the static crystal structure. We thereby suggest two hypotheses. First, the higher degree of freedom would be induced to the Ω-loop of PenL-Asn136Asp due to the loss of one hydrogen bond by the mutation that occurred outside the Ω-loop. Indeed, this effect resembles cases in other class A β-lactamase ESBLs where the stable network on Ω-loop *per se* was devastated by the substitutions at Ambler position Arg164 or Asp179 located on Ω-loop (Orencia et al., 2001; Wang et al., 2002a). In this case, the resulting enhanced flexible motion of Ω-loop may induce the transient enlargement of active site cleft and facilitate the accommodation of third-generation cephalosporins like CAZ or CTX. Second, the mutation Asn136Asp may create intrinsic dynamic conformers that would efficiently accommodate CAZ. The viewpoint of functional promiscuity in protein conformation has been suggested formerly (Tokuriki and Tawfik, 2009), whereby poorly packed or disordered conformation by an accumulation of single mutations in proteins evolves conformationally diverse structures to adapting novel substrates.

### Dynamics in Catalytic Regions of NCR-ESBLs

**FIGURE 5:**
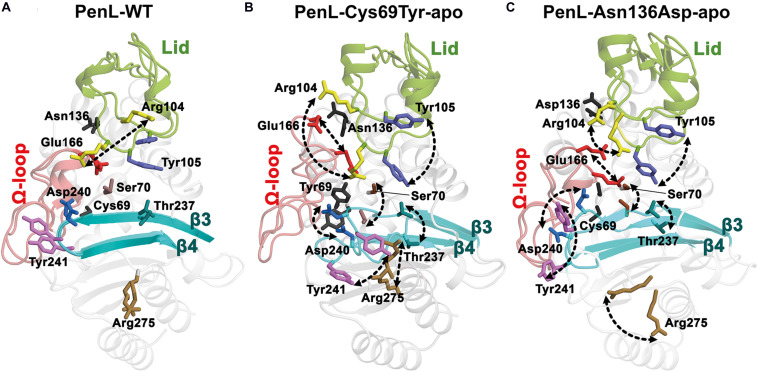
Dynamics of catalytic regions in PenL-WT and NCR-ESBLs. The dynamics of the representative residues are shown from initial to expanded state with arrows. **(A)** Dynamics of PenL-WT. Most of the residues are static during simulation except for Arg104 that fluctuates horizontally. **(B)** PenL-Cys69Try and **(C)** PenL-Asn136Asp during the 50 ns MD simulation. The dynamics of substrate binding residues and the major catalytic regions, including Ω-loop, β3-β4 strands, and lid in the NCR-ESBLs, enlarge the opening of the substrate-binding active site (see Supplementary Movie S4).

For the reasons, the molecular dynamics (MD) simulation was therefore conducted to compare the dynamic property of PenL-Asn136Asp with PenL-WT and PenL-Cys69Tyr on the CAZ binding (Figure 5 and Supplementary Figure S8). The MD simulation indicates that the overall structure of PenL-Asn136Asp was stable through a 50 ns trajectory and roughly similar to PenL-WT. However, RMSD at three critical segments of PenL-Asn136Asp, similar to PenL-Cys69Tyr, was higher than PenL-WT and appeared to fluctuate, especially at the lid region (Figure 5 and Supplementary Figure 8). Furthermore, the radius of gyration (Rgyr) plots also demonstrate a potential unfolding of two PenL-ESBLs, in contrast to the sustainable motion of PenL-WT (Figure 5). These observations strongly suggest that PenL-Asn136Asp (and PenL-Cys69Tyr) has a higher tendency for disorder than PenL-WT, which is correlated with the higher adaptability toward CAZ in term of *protein dynamism* (Tokuriki and Tawfik, 2009). In the molecular view, two regions appeared to involve in the accommodation of the large-size antibiotics followed by the transient disorder. First, the MD results showed the serial propagation of the mutation effect from Ω-loop into the β3-β4 element. The conformations of primary residues, including Asn136, Glu166, reactive Ser170, and the three essential catalytic segments involved in binding, remained unchanged in PenL-WT through MD trajectory (Figure 5A). By contrast, the mutant Asp136 side chain in PenL-Asn136Asp-apo shifted away from the Glu166 backbone of Ω-loop, leading to a large fluctuation of Glu166 (approximately 5.2 Å) and Ser70 (Figures 5B,C, Supplementary Figures S8B,C and Supplementary Movie S4). Surprisingly, the high degree of fluctuation involving the Ω-loop and Ser70 pushed the β3–β4 loop away *via* steric hindrance with a concurrent breakdown of the hydrogen bond between Asn170 and Asp240, located on the Ω-loop and β3, respectively (Figures 4B, 5C and Supplementary Movie S4). The changes in the regions, which are intensively involved in substrate recognition, may result in the momentary expansion of the active site space and the improvement of CAZ binding, likewise observed from PenL-Cys69Tyr and Ω-loop tandem repeat ESBL of PenL (Yi et al., 2016). Second, the Arg104 residue in PenL-Asn136Asp-apo penetrated the substrate-binding region compared with the Pen-WT, to facilitate the recognition and interaction with acetamido backbone of CBA through hydrogen bonding (Supplementary Figure S6B). Interestingly, the conformation of Arg104 in PenL-Asn136Asp-CBA was similar to those of PenL-Cys69Tyr-apo and -CBA, but further away than PenL-Asn136Asp-apo, suggesting that the entry of Arg104 into the substrate-binding cleft stabilized the acyl-form of PenL-Cys69Tyr *via* interaction with W5 in CBA (Figure 5C and Supplementary Figure S6). The increased affinity of PenL-Asn136Asp to CAZ also supported this result compared with PenL-WT (Table 1). Besides, Tyr105 in PenL-Asn136Asp-CBA, a well-conserved residue for ligand interaction (Papp-Wallace et al., 2013), was dislocated further way than the PenL-WT and PenL-Asn136Asp-apo (Figures 4C, 5C). Indeed, the side-chain conformation of two residues in PenL-Asn136Asp dislocated further away from the substrate-binding site in crystal structures with respect to those of PenL-Cys69-Tyr and WT (Figure 4 and Supplementary Figure S7). Therefore, the changes involving the lid region may induce momentary expansion of the active site and improve CAZ binding in the PenL-Asn136Asp and PenL-Cys69Tyr as in the canonical ESBLs (Patel et al., 2017).

## Discussion

We demonstrated that the effects of the two NCR mutations in ESBLs ultimately converged to the recognition ensemble that is one of the catalytic regions. The finding suggests a common evolutionary mechanism underlying the expansion of the substrate spectrum in β-lactamases, i.e., by widening the catalytic cleft for large-size antibiotics with high affinity *via* changes in local segments regardless of the location of mutations. Further, the electrostatic environment surrounding the oxyanion hole in PenL-Cys69Tyr can be modified by mutations enhancing substrate recognition. Although the Arg275 dislocation in PenL-Cys69Tyr accounts for the enhanced positive charge distribution around oxyanion hole, the Arg’s substantial contribution to the charge transition is still in question due to the quite remote distance (∼6.7 Å) from Thr237 on β3-β4 (Supplementary Figure S9A) and the dynamic fluctuations (Figure 5 and Supplementary Figure S8C). Of the MD simulation frames, several configurations regarding the Arg275 and Arg220 showed an augmented proximity to the active site with ∼4.0 Å and ∼4.4 Å, respectively (Supplementary Figures S9B,C). Furthermore, the disrupted regions (loop) on β3-β4 and β5-α8 (Figure 2C) by the Cys69Tyr mutation moves toward the active site as Arg275 approaches to Thr237, indicating the supply of N atoms nearer to the oxyanion hole (Supplementary Figure S8C). Therefore, the above arrangement of the basic residues and atoms in PenL-Cys69Tyr may induce a strong positive charge in the region around its oxyanion hole, which subsequently attracts the negative moiety-containing substrate, CAZ. The typical oxyanion hole, which is comprised of N atoms of reactive Ser70 and Thr237, and water W6 (Figure 2 and Supplementary Figures S5, S6), is responsible for sustaining the carbonyl group of β-lactam backbone once the β-lactam is attacked by nucleophilic Ser70. Likewise, the tetrahedral intermediate of the substrate in both CBA-bound complex structures of PenL NCR-ESBLs is undoubtedly stabilized by the typical oxyanion hole as well studied in class A β-lactamases.

In addition to Arg275, Arg104 also contributed to the oxyanion hole (Figures 3, 4). For example, Arg104 present in the lid of PenL-Cys69Tyr, protruding into the ligand-binding cleft of the enzyme together with Asn132 and W5, increased the positive charge of the oxyanion hole for further stabilization of the Michaelis complex (Supplementary Figure S7). Interestingly, the mode of interaction of Arg104 in PenL-Cys69Tyr resembled the oxyanion holes in multistep enzymes, such as thiolase (Kursula et al., 2002), which contain a “second oxyanion hole” with basic amino acids and water molecules. PenL-Asn136Asp showed relatively less perturbation of β3-β4, β5-α8, and penetration of Arg104; however, it may have transiently altered the electric potential around the oxyanion hole according to the protein dynamism theory (Tokuriki and Tawfik, 2009; Supplementary Figure S8). Indeed, MD simulations showed that Arg104 in PenL-Asn136Asp fluctuated similarly as in the PenL-Cys69Tyr (Figure 5, Supplementary Figure S8, and Supplementary Movie S4), where the flexible movement of Arg104 could momentarily modulate the electrostatic potential in the potential second oxyanion hole around substrate-binding cleft.

To the best of our knowledge, however, this potential second oxyanion hole has yet to be identified in ESBL catalysis, despite its possible existence in other ESBLs (Shimamura et al., 2002; Chen et al., 2005). The existence of the potential second oxyanion hole in the ESBLs may be attributed to their kinetic behaviors (Merilainen et al., 2009), as well as increased affinity. In other words, the increased affinity of PenL-Cys69Tyr for CAZ may facilitate the acylation step but simultaneously attenuate the deacylation process due to the formation of a stable acyl-enzyme complex, in which Arg104 on the flexible loop could be discharged from the interaction with Asn132 and W6 and subsequently could disrupt the potential “second oxyanion hole” before releasing a product (Figure 3 and Supplementary Figure S6). The precise hydrolytic mechanism of CAZ mediated by NCR-ESBLs, however, needs to be further investigated.

In summary, we have described the structural mechanism underlying the hydrolysis of third-generation cephalosporins by novel NCR-ESBLs. Although the mutations were unconventional in that they occurred outside of the catalytic region for hydrolysis and substrate binding, the class A NCR-ESBLs exhibited altered substrate specificity and carried an expanded active site similar to the canonical class A ESBLs. We believe that the novel substrate-spectrum expansion mechanism of the class A β-lactamases described in this study will significantly enhance the current knowledge of the evolutionary trends of ESBLs against new antibiotics. Furthermore, from a practical perspective, such information will be crucial for developing novel and useful inhibitors or antibiotics targeting various class A ESBLs, including emerging NCR-ESBLs.

## Data Availability Statement

The datasets presented in this study can be found in online repositories. The names of the repository/repositories and accession number(s) can be found in the article/Supplementary Material.

## Author Contributions

TC and HY performed the experiments and generated the data for this study with an equal contribution. ID, SG, and JC helped with kinetics, CD, molecular dynamics analysis, generating figures, and crystallization. KHL and SR helped design and validation of molecular dynamics results with critical comments. HSK and SHL developed the ideas, designed the experiments, analyzed the data, and prepared the manuscript. All authors contributed to the article and approved the submitted version.

## Conflict of Interest

The authors declare that the research was conducted in the absence of any commercial or financial relationships that could be construed as a potential conflict of interest.

## Footnotes

1 https://www.lahey.org/Studies/

## References

[B1] Abergel C. (2013). Molecular replacement: tricks and treats. *Acta Crystallogr. D Biol. Crystallogr.* 69(Pt 11) 2167–2173. 10.1107/S0907444913015291 24189227 PMC3817689

[B2] Abraham M. J. Gready J. E. (2011). Optimization of parameters for molecular dynamics simulation using smooth particle−mesh Ewald in GROMACS 4.5. *J. Comput. Chem.* 32 2031–2040. 10.1002/jcc.21773 21469158

[B3] Afonine P. V. Grosse-Kunstleve R. W. Echols N. Headd J. J. Moriarty N. W. Mustyakimov M. (2012). Towards automated crystallographic structure refinement with phenix.refine. *Acta Crystallogr. D Biol. Crystallogr.* 68(Pt 4) 352–367. 10.1107/S0907444912001308 22505256 PMC3322595

[B4] Banerjee S. Pieper U. Kapadia G. Pannell L. K. Herzberg O. (1998). Role of the ¿-loop in the activity, substrate specificity, and structure of class A β-lactamase. *Biochemistry* 37 3286–3296. 9521648 10.1021/bi972127f

[B5] Banerjee S. Shigematsu N. Pannell L. K. Ruvinov S. Orban J. Schwarz F. (1997). Probing the non-proline Cis peptide bond in β-Lactamase from *Staphylococcus aureus* PC1 by the Replacement Asn136 → Ala. *Biochemistry* 36 10857–10866. 10.1021/bi970352r 9283075

[B6] Berg J. M. Tymoczko J. L. Stryer L. (2012). *Biochemistry.* Kate Ahr Parker: W. H. Freeman and Company.

[B7] Bethel C. R. Hujer A. M. Hujer K. M. Thomson J. M. Ruszczycky M. W. Anderson V. E. (2006). Role of Asp104 in the SHV -Lactamase. *Antimicrob. Agents Chemother.* 50 4124–4131. 10.1128/aac.00848-06 16982784 PMC1694000

[B8] Brown N. G. Shanker S. Prasad B. V. Palzkill T. (2009). Structural and biochemical evidence that a TEM-1 beta-lactamase N170G active site mutant acts via substrate-assisted catalysis. *J. Biol. Chem.* 284 33703–33712. 10.1074/jbc.M109.053819 19812041 PMC2785212

[B9] Celenza G. Luzi C. Aschi M. Segatore B. Setacci D. Pellegrini C. (2008). Natural D240G Toho-1 mutant conferring resistance to ceftazidime: biochemical characterization of CTX-M-43. *J. Antimicrob. Chemother.* 62 991–997. 10.1093/jac/dkn339 18755695

[B10] Chaibi E. B. Peduzzi J. Farzaneh S. Barthelemy M. Sirot D. Labia R. (1998). Clinical inhibitor-resistant mutants of the beta-lactamase TEM-1 at amino-acid position 69. Kinetic analysis and molecular modelling. *Biochim. Biophys. Acta* 1382 38–46. 10.1016/s0167-4838(97)00127-1 9507060

[B11] Chen Y. Shoichet B. Bonnet R. (2005). Structure, function, and inhibition along the reaction coordinate of CTX-M beta-lactamases. *J. Am. Chem. Soc.* 127 5423–5434. 10.1021/ja042850a 15826180 PMC1360657

[B12] Dance D. (2014). Treatment and prophylaxis of melioidosis. *Int. J. Antimicrob. Agents* 43 310–318. 10.1016/j.ijantimicag.2014.01.005 24613038 PMC4236584

[B13] Dobson R. Yi H. Cho K.-H. Cho Y. S. Kim K. Nierman W. C. (2012). Twelve positions in a β-lactamase that can expand its substrate spectrum with a single amino acid substitution. *PLoS One* 7:e37585. 10.1371/journal.pone.0037585 22629423 PMC3358254

[B14] Doucet N. De Wals P.-Y. Pelletier J. N. (2004). Site-saturation mutagenesis of Tyr-105 reveals its importance in substrate stabilization and discrimination in TEM-1 β-Lactamase. *J. Biol. Chem.* 279 46295–46303. 10.1074/jbc.M407606200 15326193

[B15] Drawz S. M. Bonomo R. A. (2010). Three decades of beta-lactamase inhibitors. *Clin. Microbiol. Rev.* 23 160–201. 10.1128/CMR.00037-09 20065329 PMC2806661

[B16] Du Bois S. K. Marriott M. S. Amyes S. G. (1995). TEM- and SHV-derived extended-spectrum beta-lactamases: relationship between selection, structure and function. *J. Antimicrob. Chemother.* 35 7–22. 10.1093/jac/35.1.7 7768784

[B17] Echols N. Moriarty N. W. Klei H. E. Afonine P. V. Bunkoczi G. Headd J. J. (2014). Automating crystallographic structure solution and refinement of protein-ligand complexes. *Acta Crystallogr. D Biol. Crystallogr.* 70(Pt 1) 144–154. 10.1107/S139900471302748X 24419387 PMC3919266

[B18] Emsley P. Cowtan K. (2004). Coot: model-building tools for molecular graphics. *Acta Crystallogr. D Biol. Crystallogr.* 60(Pt 12) 2126–2132. 10.1107/S0907444904019158 15572765

[B19] Giakkoupi P. Hujer A. M. Miriagou V. Tzelepi E. Bonomo R. A. Tzouvelekis L. S. (2001). Substitution of Thr for Ala-237 in TEM-17, TEM-12 and TEM-26: alterations in β-lactam resistance conferred on *Escherichia coli*. *FEMS Microbiol. Lett.* 201 37–40. 10.1016/s0378-1097(01)00239-7 11445164

[B20] Giakkoupi P. Miriagou V. Gazouli M. Tzelepi E. Legakis N. J. Tzouvelekis L. S. (1998). Properties of mutant SHV-5 beta-lactamases constructed by substitution of isoleucine or valine for methionine at position 69. *Antimicrob. Agents Chemother.* 42 1281–1283. 10.1128/aac.42.5.1281 9593168 PMC105805

[B21] Gniadkowski M. (2001). Evolution and epidemiology of extended-spectrum beta-lactamases (ESBLs) and ESBL-producing microorganisms. *Clin. Microbiol. Infect.* 7 597–608. 10.1046/j.1198-743x.2001.00330.x 11737084

[B22] Guillaume G. Vanhove M. Lamotte-Brasseur J. Ledent P. Jamin M. Joris B. (1997). Site-directed mutagenesis of glutamate 166 in Two b-Lactamases. *J. Biol. Chem.* 272 5438–5444. 10.1074/jbc.272.9.5438 9038144

[B23] Helfand M. S. Hujer A. M. Sönnichsen F. D. Bonomo R. A. (2002). Unexpected advanced generation cephalosporinase activity of the M69F variant of SHV β-Lactamase. *J. Biol. Chem.* 277 47719–47723. 10.1074/jbc.M207271200 12354765

[B24] Huletsky A. Knox J. R. Levesque R. C. (1993). Role of Ser-238 and Lys-240 in the hydrolysis of third-generation cephalosporins by SHV-type beta-lactamases probed by site-directed mutagenesis and three-dimensional modeling. *J. Biol. Chem.* 268 3690–3697. 8429044

[B25] Hunter J. D. (2007). Matplotlib: a 2D graphics environment. *Comput. Sci. Eng.* 9 90–95.

[B26] Kursula P. Ojala J. Lambeir A. M. Wierenga R. K. (2002). The catalytic cycle of biosynthetic thiolase: a conformational journey of an acetyl group through four binding modes and two oxyanion holes. *Biochemistry* 41 15543–15556. 10.1021/bi0266232 12501183

[B27] Lamotte-Brasseur J. Dive G. Dideberg O. Charlier P. Frere J. M. Ghuysen J. M. (1991). Mechanism of acyl transfer by the class A serine beta-lactamase of *Streptomyces albus* G. *Biochem. J.* 279(Pt 1) 213–221. 10.1042/bj2790213 1930139 PMC1151568

[B28] Lemkul J. A. Roux B. van der Spoel D. MacKerell A. D. Jr. (2015). Implementation of extended Lagrangian dynamics in GROMACS for polarizable simulations using the classical Drude oscillator model. *J. Comput. Chem.* 36 1473–1479. 10.1002/jcc.23937 25962472 PMC4481176

[B29] Levitt P. S. Papp-Wallace K. M. Taracila M. A. Hujer A. M. Winkler M. L. Smith K. M. (2012). Exploring the role of a conserved class a residue in the Ω-Loop of KPC-2 β-Lactamase. *J. Biol. Chem.* 287 31783–31793. 10.1074/jbc.M112.348540 22843686 PMC3442512

[B30] Merilainen G. Poikela V. Kursula P. Wierenga R. K. (2009). The thiolase reaction mechanism: the importance of Asn316 and His348 for stabilizing the enolate intermediate of the Claisen condensation. *Biochemistry* 48 11011–11025. 10.1021/bi901069h 19842716

[B31] Meroueh S. O. Fisher J. F. Schlegel H. B. Mobashery S. (2005). Ab Initio QM/MM study of class A b-Lactamase acylation: dual participation of Glu166 and Lys73 in a concerted base promotion of Ser70. *J. Am. Chem. Soc.* 127 15397–15407. 10.1021/ja051592u 16262403

[B32] Meroueh S. O. Roblin P. Golemi D. Maveyraud L. Vakulenko S. B. Zhang Y. (2002). Molecular dynamics at the root of expansion of function in the M69L inhibitor-resistant TEM b-lactamase from *Escherichia coli*. *J. Am. Chem. Soc.* 124 9422–9430. 10.1021/ja026547q 12167037

[B33] Murshudov G. N. Vagin A. A. Dodson E. J. (1997). Refinement of macromolecular structures by the maximum-likelihood method. *Acta Crystallogr. D Biol. Crystallogr.* 53(Pt 3) 240–255. 10.1107/S0907444996012255 15299926

[B34] Oliphant T. E. (2007). Python for scientific computing. *Comput. Sci. Eng.* 9 10–20. 10.1109/Mcse.2007.58

[B35] Orencia M. C. Yoon J. S. Ness J. E. Stemmer W. P. Stevens R. C. (2001). Predicting the emergence of antibiotic resistance by directed evolution and structural analysis. *Nat. Struct. Biol.* 8 238–242. 10.1038/84981 11224569

[B36] Page M. G. P. (2008). Extended-spectrum β-lactamases: structure and kinetic mechanism. *Clin. Microbiol. Infect.* 14 63–74. 10.1111/j.1469-0691.2007.01863.x 18154529

[B37] Palzkill T. (2018). Structural and mechanistic basis for extended-spectrum drug-resistance mutations in altering the specificity of TEM, CTX-M, and KPC beta-lactamases. *Front. Mol. Biosci.* 5:16. 10.3389/fmolb.2018.00016 29527530 PMC5829062

[B38] Papp-Wallace K. M. Taracila M. Hornick J. M. Hujer A. M. Hujer K. M. Distler A. M. (2010). Substrate selectivity and a novel role in inhibitor discrimination by residue 237 in the KPC-2 beta-lactamase. *Antimicrob. Agents Chemother.* 54 2867–2877. 10.1128/AAC.00197-10 20421396 PMC2897288

[B39] Papp-Wallace K. M. Taracila M. A. Gatta J. A. Ohuchi N. Bonomo R. A. Nukaga M. (2013). Insights into beta-lactamases from Burkholderia species, two phylogenetically related yet distinct resistance determinants. *J. Biol. Chem.* 288 19090–19102. 10.1074/jbc.M113.458315 23658015 PMC3696682

[B40] Papp-Wallace K. M. Taracila M. A. Smith K. M. Xu Y. Bonomo R. A. (2012). Understanding the molecular determinants of substrate and inhibitor specificities in the Carbapenemase KPC-2: exploring the roles of Arg220 and Glu276. *Antimicrob. Agents Chemother.* 56 4428–4438. 10.1128/AAC.05769-11 22687511 PMC3421566

[B41] Patel M. P. Hu L. Stojanoski V. Sankaran B. Prasad B. V. V. Palzkill T. (2017). The drug-resistant variant P167S expands the substrate profile of CTX-M beta-Lactamases for Oxyimino-Cephalosporin Antibiotics by Enlarging the Active Site upon Acylation. *Biochemistry* 56 3443–3453. 10.1021/acs.biochem.7b00176 28613873 PMC5645026

[B42] Perez F. Endimiani A. Hujer K. M. Bonomo R. A. (2007). The continuing challenge of ESBLs. *Curr. Opin. Pharmacol.* 7 459–469. 10.1016/j.coph.2007.08.003 17875405 PMC2235939

[B43] Petit A. Maveyraud L. Lenfant F. Samama J. P. Labia R. Masson J. M. (1995). Multiple substitutions at position 104 of b-lactamase TEM-1: assessing the role of this residue in substrate specificity. *Biochem. J.* 305 33–40. 10.1042/bj3050033 7826350 PMC1136426

[B44] Ramos M. C. Horta V. A. C. Horta B. A. C. (2019). Molecular dynamics simulations of PAMAM and PPI dendrimers using the GROMOS-Compatible 2016H66 Force Field. *J. Chem. Inform. Model.* 59 1444–1457. 10.1021/acs.jcim.8b00911 30875214

[B45] Ruggiero M. Curto L. Brunetti F. Sauvage E. Galleni M. Power P. (2017). Impact of Mutations at Arg220 and Thr237 in PER-2 β-lactamase on conformation, activity, and susceptibility to inhibitors. *Antimicrob. Agents Chemother.* 61 e02193-16. 10.1128/aac.02193-16 28320728 PMC5444140

[B46] Sam I. C. See K. H. Puthucheary S. D. (2009). Variations in ceftazidime and amoxicillin-clavulanate susceptibilities within a clonal infection of *Burkholderia pseudomallei*. *J. Clin. Microbiol.* 47 1556–1558. 10.1128/JCM.01657-08 19297597 PMC2681875

[B47] Shimamura T. Ibuka A. Fushinobu S. Wakagi T. Ishiguro M. Ishii Y. (2002). Acyl-intermediate structures of the extended-spectrum class A beta-lactamase, Toho-1, in complex with cefotaxime, cephalothin, and benzylpenicillin. *J. Biol. Chem.* 277 46601–46608. 10.1074/jbc.M207884200 12221102

[B48] Shimizu-Ibuka A. Oishi M. Yamada S. Ishii Y. Mura K. Sakai H. (2011). Roles of residues Cys69, Asn104, Phe160, Gly232, Ser237, and Asp240 in extended-spectrum β-Lactamase Toho-1. *Antimicrob. Agents Chemother.* 55 284–290. 10.1128/aac.00098-10 21078949 PMC3019690

[B49] Sirot D. Recule C. Chaibi E. B. Bret L. Croize J. Chanal-Claris C. (1997). A complex mutant of TEM-1 b-Lactamase with mutations encountered in both IRT-4 and extended-spectrum TEM-15 produced by an *Escherichia coli* clinical isolate. *Antimicrob. Agents Chemother.* 41 1322–1325. 10.1128/aac.41.6.1322 9174192 PMC163908

[B50] Tokuriki N. Tawfik D. S. (2009). Protein dynamism and evolvability. *Science* 324 203–207. 10.1126/science.1169375 19359577

[B51] Totir M. A. Padayatti P. S. Helfand M. S. Carey M. P. Bonomo R. A. Carey P. R. (2006). Effect of the inhibitor-resistant M69V substitution on the structures and populations of trans-enamine beta-lactamase intermediates. *Biochemistry* 45 11895–11904. 10.1021/bi060990m 17002290 PMC2596060

[B52] Wang X. Minasov G. Shoichet B. K. (2002a). Evolution of an antibiotic resistance enzyme constrained by stability and activity trade-offs. *J. Mol. Biol.* 320 85–95. 10.1016/S0022-2836(02)00400-X 12079336

[B53] Wang X. Minasov G. Shoichet B. K. (2002b). The structural bases of antibiotic resistance in the clinically derived mutant β-Lactamases TEM-30, TEM-32, and TEM-34. *J. Biol. Chem.* 277 32149–32156. 10.1074/jbc.M204212200 12058046

[B54] Yi H. Choi J. M. Hwang J. Prati F. Cao T.-P. Lee S. H. (2016). High adaptability of the omega loop underlies the substrate-spectrum-extension evolution of a class A β-lactamase, PenL. *Sci. Rep.* 6:36527. 10.1038/srep36527 27827433 PMC5101513

