# Supplementary material for: Non-catalytic-Region Mutations Conferring Transition of Class A β-Lactamases Into ESBLs
Source: Front Mol Biosci. 2020 Nov 27;7:598998. doi: 10.3389/fmolb.2020.598998 (PMC7737660; doi:10.3389/fmolb.2020.598998)
Supplement: Supplementary file 1 [file Table_1.DOCX]

Non-Catalytic-Region Mutations Conferring Transition of Class A β-Lactamases into ESBLs

Running title: Structural analysis of NCR-ESBLs

Thinh-Phat Cao^1,3,†^**,** Hyojeong Yi^2,†^, Immanuel Dhanasingh^1^,Suparna Ghosh^1^, Jin Myung Choi^1^, Kun Ho Lee^3,4^, Seol Ryu^5^, Heenam Stanley Kim^2,^*, and Sung Haeng Lee^1,3,^*

^1^ Department of Cellular and Molecular Medicine, Chosun University School of Medicine, Gwangju 61452, Republic of Korea

^2^ Division of Biosystems & Biomedical Sciences, College of Health Sciences, Korea University, 145 Anam-ro, Seongbuk-Gu, Seoul 02841, Republic of Korea

^3^ Gwangju Alzheimer’s disease and Related Dementia Cohort Research Center & Department of Biomedical Sciences, College of Natural Sciences and Public Health and Safety, Chosun University, Gwangju 61452, Republic of Korea

^4^ Aging Neuroscience Research Group, Korea Brain Research Institute, Daegu 41062, Republic of Korea

^5^ Department of Chemistry, Chosun University, Gwangju 61452, Republic of Korea

*Correspondence:

Sung Haeng Lee

sunglee@chosun.ac.kr

Heenam Stanley Kim

hstanleykim@korea.ac.kr

^†^These authors contributed equally to this work.

***Supplementary Information***

**MATERIALS AND METHODS**

**Determination of kinetic**

The initial velocities of PenL-WT, PenL-Cys69Tyr, and PenL-Asn136Asp were measured using a common procedure during the first 10 s and the velocities (*v*) were fitted to Michealis-Menten equation, as follows:

$$v= \frac{k_{cat}E_{T}\left[ S \right]}{K_{M}+ \left[ S \right]} \left( 1 \right),$$

where $k_{cat}$ is the turnover number, *K*_M_ denotes Michaelis constant, [*S*] refers to substrate concentration, and $E_{T}$ is the total enzyme concentration in the reaction.

**Circular dichroism spectra**

Circular dichroism (CD) spectra in the far-UV region (190 – 250 nm) were monitored with a Jasco J-1500 spectropolarimeter at 25°C using a Peltier temperature controller. An aliquot of 10 μM of protein in buffer containing 20 mM Tris-H_2_SO_4_ and 50 mM NaF, at pH 7.5 was transferred to a cuvette with 100 mm path length. The first baseline was determined using the same buffer. The baseline for CBA was initially corrected with the first baseline, which was in turn used for protein-ligand experiment subtraction. A triplicate scan was conducted for each data set with 0.1 nm intervals and 1 mm bandwidth, and then averaged. Protein-ligand experiments were conducted by adding 25 μM of CBA to the protein sample (10 μM) followed by incubation for 1 hour at room temperature.

Electrostatic potential calculation

The electrostatic distribution on the surface of proteins was calculated using Adaptive Poisson-Boltzmann Solver, or APBS([Jurrus E et al., 2018](#_ENREF_6)). The APBS plugin is available as a PyMOL incentive version 2.0. The protein models in *.pdb format were prepared using PDB2PQR,([Dolinsky TJ et al., 2004](#_ENREF_2)) which adds hydrogens and missing side chain atoms, and assigns partial charges and radii initially. The electrostatic parameters were calculated under default ionic strength condition (0.15) at 300 K. The pH of all calculations was firmly fixed at 7.0 and the corresponding pK_a_ values of particles were computed using Propka([Olsson et al., 2011](#_ENREF_8)).

Electrostatic potential was expressed as k_b_T/e_c_, or kT/e in short, where k_b_ (or k) is the Boltzmann constant (1.3806504 × 10^−23^ J K^-1^), T denotes the calculated temperature (300 K), and e_c_ (or e) refers to the charge of an electron (1.60217646 × 10^-19^ C). The electrostatic potential of all the computed proteins in this study was color-coded from -3000 kT/e (red) to zero (white) to 3000 kT/e (blue), and visualized in PyMOL.

**RESULTS AND DISCUSSION**

**Mutational hotspots in the canonical ESBLs of class A β-lactamases**

In general, class A β-lactamases regardless of subfamilies share similar overall architecture as shown in Figure S1 below, in which the residues of catalytic ensemble (black dots) including reactive Ser70 at the center are surrounded by the recognition ensemble consisting of three critical segments including Ω-loop (red), lid (green), and strands β3-β4 (blue). These three segments are known to directly mediate substrate recognition and hydrolysis. The mutations triggering the emergence of ESBLs in TEM- and SHV-type class A β-lactamases are listed at the following URL: <https://www.lahey.org/Studies/>. A review of this database revealed that the emergence of ESBLs is triggered primarily by single substitutions involving residues in one of the three segments. The positions of such residues are indicated by spheres numbered in yellow in Figure S1. Multiple substitutions may occur incidentally reflecting the extended spectrum, for instance, double substitutions of Arg164Ser and Glu240Lys in TEM-10([J P Quinn et al., 1989](#_ENREF_5)), or triple substitutions of Arg164Ser, Glu240Lys and Glu104Lys in TEM-46([C Chanal-Claris et al., 1997](#_ENREF_1)). In addition to multiple substitution mutations in the three critical segments (recognition ensemble), another type of single mutation may contribute to the structural stability of the enzyme following the emergence of ESBLs. Most notably, the mutation at residue 182, originally occupied by a threonine, was located behind the active site. In the structural study of TEM-52 (Glu104Lys, Gly238Ser, and Thr182Met), the substitution Thr182Met may have further stabilized the enzyme structure([M. Cecilia Orencia et al., 2001](#_ENREF_7)). Based on the list at <https://www.lahey.org/Studies/>, another frequent and unusual mutation site with an ambiguous role was identified at Gln39 (in TEM-type) or Leu35 (in SHV-type), which was distant from the active site and mostly exposed to the surface. We assumed that the mutation at this site was a random variation during evolution, with an insignificant role.

**
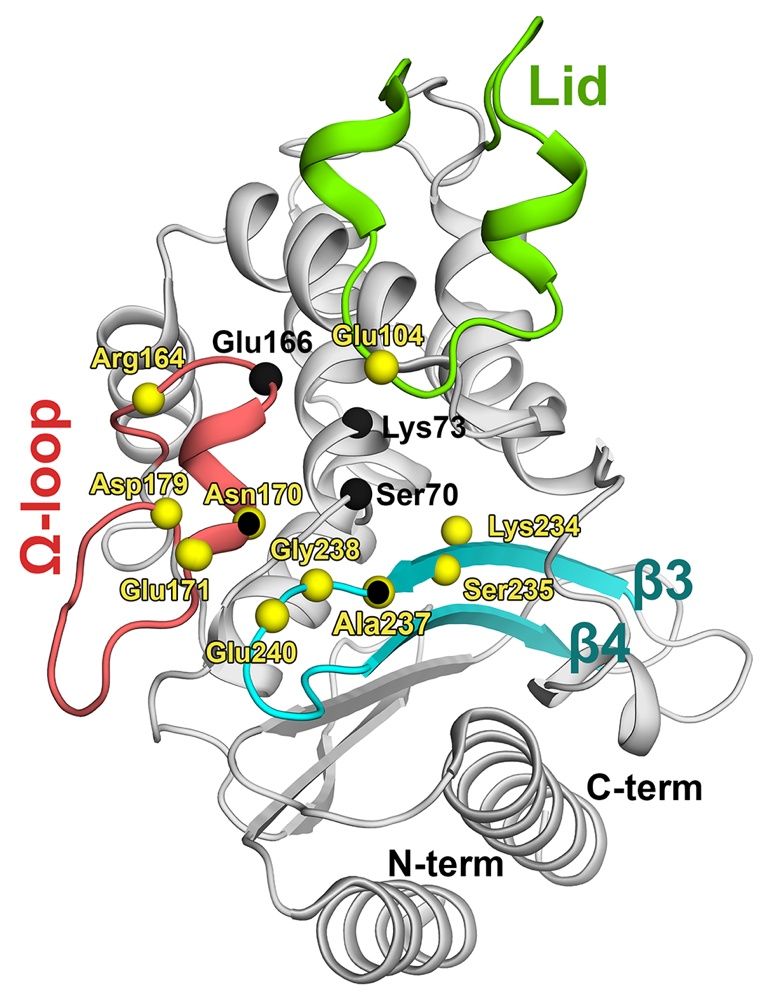
**

**Figure S1. Cartoon representation of** β**-lactamase with emphasis on the catalytic region (catalytic and recognition ensemble).** Mutational hotspots occurring in the three essential segments induce the emergence of ESBLs in class A β-lactamase represented in the TEM-1 structure (PDB ID: 1M40) ([George Minasov et al., 2002](#_ENREF_4)). The double-colored dots represent the residues (170 and 237) of the mutational hotspots (yellow) among catalytic amino acids (black).

**Kinetic analysis of PenL-Cys69Tyr and PenL-Asn136Asp**

In principle, β-lactam hydrolysis occurs generally in three major steps as reported previously([Palzkill, 2018](#_ENREF_9)), and as shown in Scheme 1 below.

*K_M_*

*k_cat_*

The hydrolysis of β-lactam backbone is conserved in all class A β-lactamases([Drawz and Bonomo, 2010](#_ENREF_3); [Papp-Wallace et al., 2013](#_ENREF_11)). The increased hydrolysis of the novel substrate, i.e. CAZ in this case, is thus accomplished *via* specific enzyme modifications in the first step involving substrate binding. Such modifications ultimately affect the substrate affinity (or *K*_M_ value), and the reaction rate (or *k*_cat_ value). Therefore, the kinetic study was performed to elucidate the role of two single-amino acid substitutions in substrate recognition by the enzyme.

Due to poor spectroscopic properties of CAZ, the catalytic performance of PenL-WT at CAZ concentrations greater than 100 μM cannot be monitored. The initial velocity of PenL-WT was measured during the first 10 s (Figure S2), and the velocities (*v*) were fitted to the Michaelis-Menten equation (Figure 1B). Therefore, the PenL-WT hydrolyzed CAZ with the first-order kinetics (Figure 1B), and its kinetic parameters were determined by the reciprocal plotting. The *K*_M_ and *k*_cat_ for PenL-WT were very high, and the catalytic efficiency (*k*_cat_*/K*_M_) estimated from the slope of the regression curve was ~3.0 nM^-1^s^-1^ (Table 1). Therefore, the CAZ affinity of PenL-WT was very low and the rate of CAZ hydrolysis depended strongly on substrate concentration, to prevent bacterial growth.


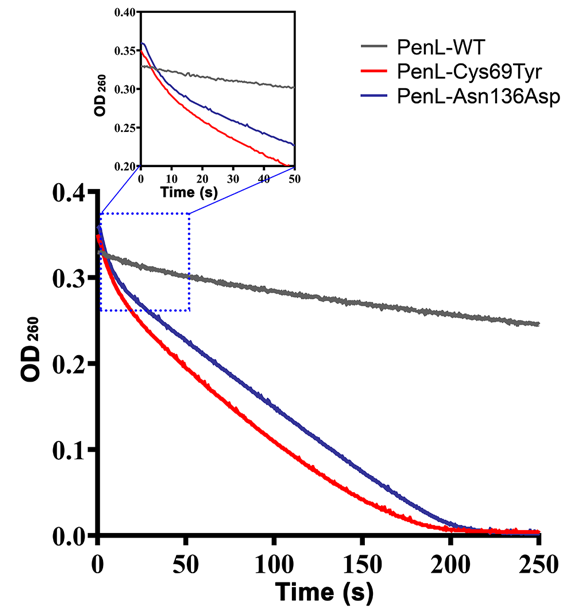


**Figure S2. CAZ hydrolysis by NCR-ESBLs.** PenL-WT, PenL-Cys69Tyr and PenL-Asn136Asp catalyzed the reaction in time-dependent manner at the representative concentration of CAZ (25 μM). The measurements of rate were carried out during the first 10 s, which is linearly related to CAZ decay (magnified box)

By contrast, the rate of CAZ hydrolysis in the two PenL variants rapidly reached maximum velocity in CAZ concentration-dependent manner (Figure 1B). As a result, the *K*_M_ for two PenL variants was significantly reduced compared with PenL-WT (Table 1), indicating enhanced CAZ affinity in the active site of the variants Cys69Tyr and Asn136Asp. Furthermore, the catalytic efficiency *k*_cat_*/K*_M_ of the two PenL variants was also increased to 3~5 folds compared with PenL-WT (Table 1). Moreover, because PenL-Cys69Tyr and PenL-Asn136Asp showed a high affinity toward CAZ, a stable acyl-enzyme complex may be formed after binding (Scheme 1).

**CD spectral analysis**

Because the catalytic activity of an enzyme is firmly correlated with its conformational integrity([Rigos et al., 2006](#_ENREF_12)), the distinct kinetic properties of the two PenL variants suggest that the mutations alter the substrate recognition, which leads to increased substrate affinity. Such alteration may induce changes in enzyme conformation under the effect of single amino acid substitutions. Further, a prior study analyzing PenI-Cys69Phe homologies also suggested two different conformations of the variant, one of which induced rapid CAZ hydrolysis([Papp-Wallace et al., 2016](#_ENREF_10)). It is tempting to expect that PenL-Cys69Tyr, and even PenL-Asn136Asp show such characteristic features. Therefore, to elucidate the conformational changes of the two PenL variants compared with PenL-WT in solution (at 25°C), the circular dichroism (CD) spectra were analyzed (Figure S3). Interestingly, the spectra of apo-form of the two PenL variants were similar to those of PenL-WT (Figure S3, solid lines), suggesting conformational similarity with the wild-type enzyme. However, in the presence of CBA, the spectra of two PenL variants deviated in both α (~208 and 220 nm) and β (~198 nm) content, while the spectrum of PenL-WT was probably attributed to empirical error (Figure S3A, dashed lines). The concentration of CBA in the reaction (25 μM) shifted the *K*_M_ value of the two PenL variants toward CAZ (Table 1), indicating the reliability of deviation between the spectra of two PenL variants with and without CBA. Nonetheless, such CBA concentration may be significantly lower than the *K*_M_ value of PenL-WT, for which the deviation was undetected. Overall, the CD spectra suggest that the alternative conformation of PenL-Cys69Tyr and PenL-Asn136Asp was induced by substrate binding, which further supports the hypothesis that the two variants recognize CAZ via a different mechanism compared with the wild-type enzyme. In CD experiments with substrate CAZ, the spectral changes are also similar to those from CBA (Figure S3B). Moreover, the deviations in CD spectral change between the two ESBLs were even less than those with CBA, which might result from two possible scenarios, the rapid hydrolysis of CAZ accompanied by the slight conformational changes.

**
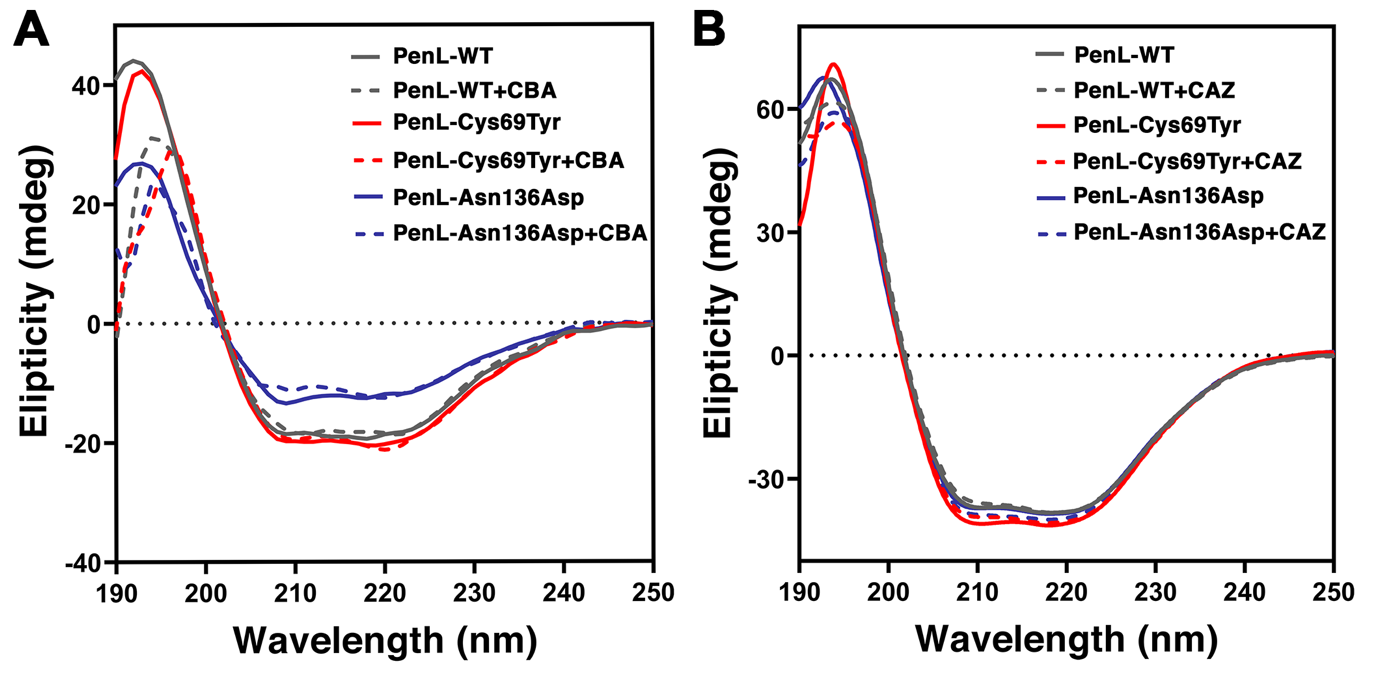
**

**Figure S3. CD spectra analysis of PenL-WT and two NCR-ESBLs**

**Crystal structure analysis**

We determined the crystal structures of two PenL variants at 1.3 and 1.4 Å, respectively (Table S1). The global structure of PenL-Cys69Tyr and PenL-Asn136Asp was almost identical to that of the wild-type enzyme, with RMSDs of 0.221 and 0.186 Å at Cα atom, respectively, compared with PenL-WT (PDB ID: 5GL9)([Yi et al., 2016](#_ENREF_13)) (Figure S4). Also, the three-dimensional arrangement of residues around the catalytic region in the apo- forms of PenL-WT and the two NCR-ESBLs are well overlapped, indicating the conservation of hydrolysis capability of β-lactam of antibiotics (Figure S5 and Figure 2A). Interestingly, the electrostatic distribution of NCR-ESBL-apo forms appears differently around the catalytic region, where oxyanion hole region becomes more positive charged in comparison with that of WT. In particular, the electro-positivity around oxyanion hole in PenL-Cys69Tyr NCR-ESBL is increased more than PenL-Asn136Asp and PenL-WT, indicating that the ligand-binding region of NCR-ESBLs may have a higher affinity to large-sized CAZ containing additional hydroxyl groups. However, the positive electrostatic nature in the catalytic region and oxyanion hole of PenL-Asn136Asp NCR-ESBL has either similar to its WT or less positive charge than that of PenL-Cys69Tyr NCR-ESBL (Figure S4, S6). Instead, the size of the ligand-binding cavity of PenL-Cys69Tyr NCR-ESBL is also expanded large enough to accept and dock the cephalosporin CAZ. Therefore, it may suggest that the novel two NCR-ESBLs either or both change the size of catalytic cavity and the electrostatic nature of oxyanion hole, through changing of conformations of the three essential catalytic regions but keeping the integrity of hydrolysis-involving residues, to accept the large and more hydrophilic third-generation cephalosporin antibiotics (Figures 2, 4, S4, S6, S7, and S8)


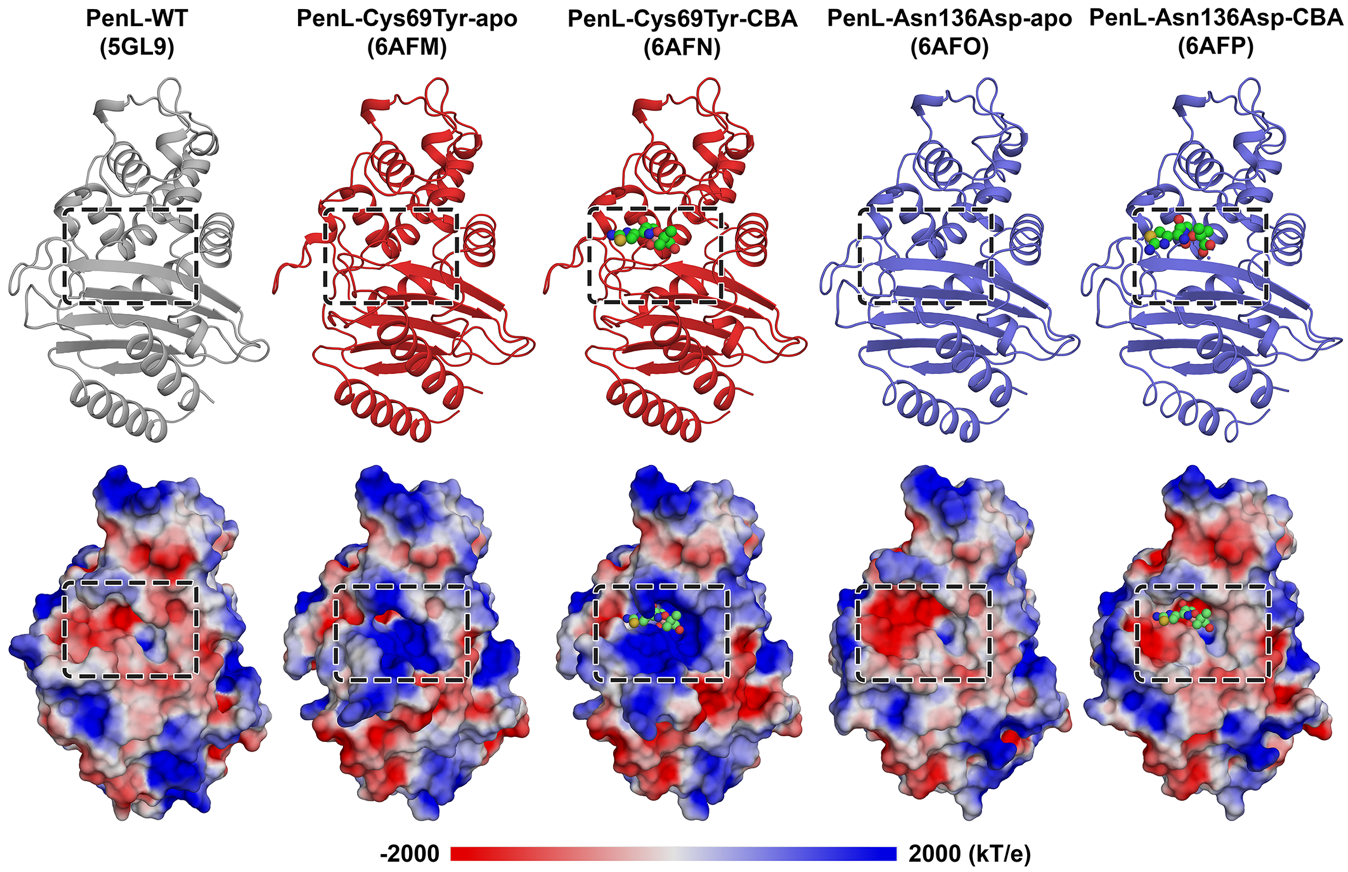


**Figure S4. Overall structures of PenL-Cys69Tyr and PenL-Asn136Asp compared with PenL-WT.** Crystal structures of PenL-Cys69Tyr and PenL-Asn136Asp are shown in comparison with PenL-WT (5GL9). The corresponding electrostatic distribution on the surface is displayed in the same column. The active-site cleft is marked with dashed-line rounded rectangles.


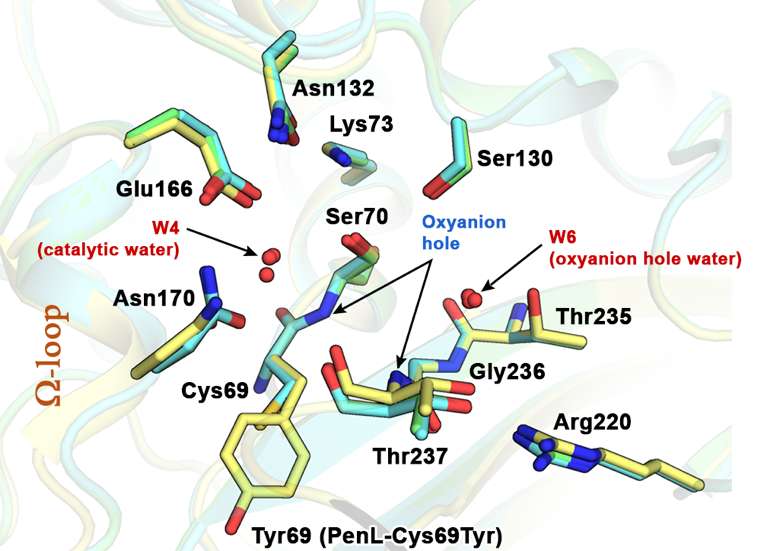


**Figure S5. Conserved active site residues (catalytic ensemble) of the PenL-Cys69Tyr-apo ESBL variant and PenL-WT.** Residues in the catalytic ensemble are numbered according to Ambler system. This ensemble is strictly conserved among class A *β*-lactamases.


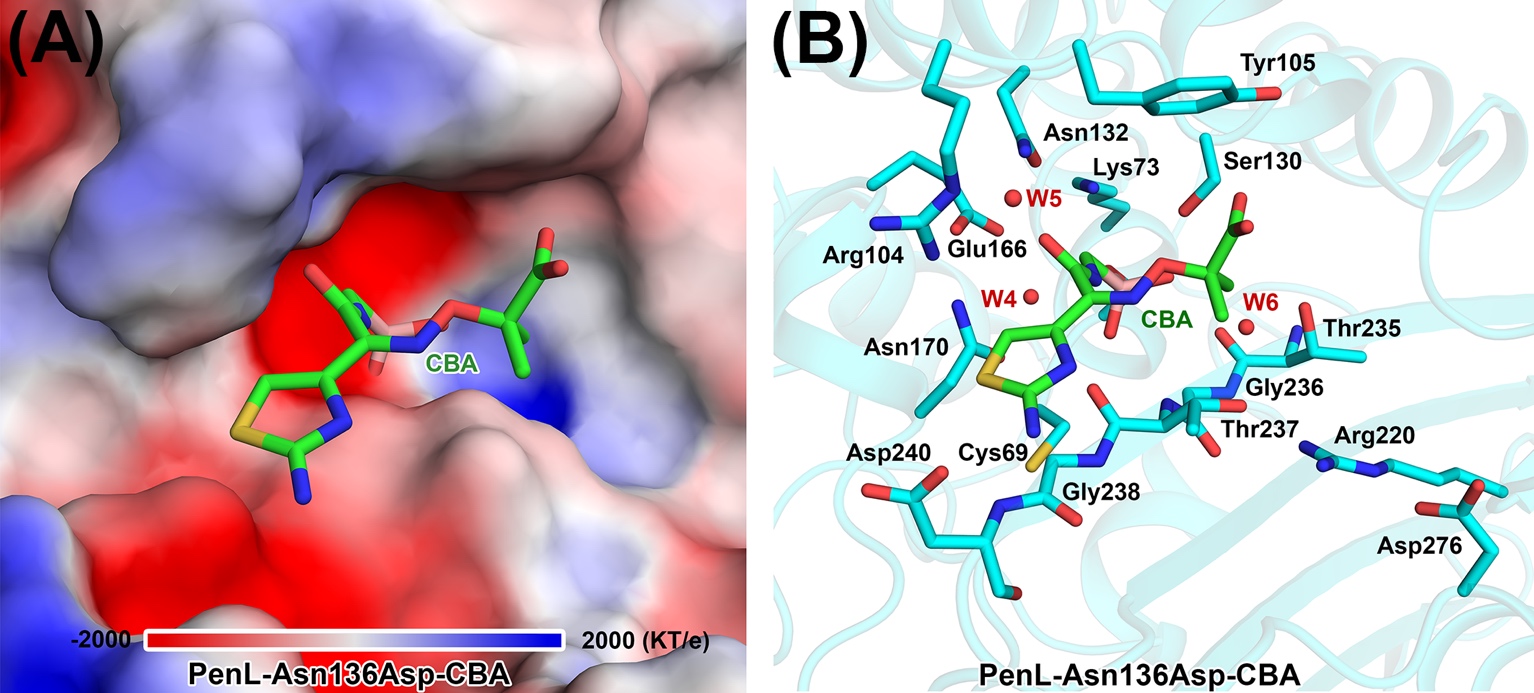


**Figure S6. CBA-bound structure of PenL-Asn136Asp.** CBA-bound forms of PenL-Asn136Asp was obtained. The CBA acylation form of PenL-Asn136Asp is similar to that of PenL-Cys69Tyr (see Figure 2). (A) Electrostatic distribution is also shown in the same angle view. (B) The residues on β3 mainly interact with CBA, and Arg104 and Tyr105 underwent conformational changes in between apo- and CBA-bound structure.

**
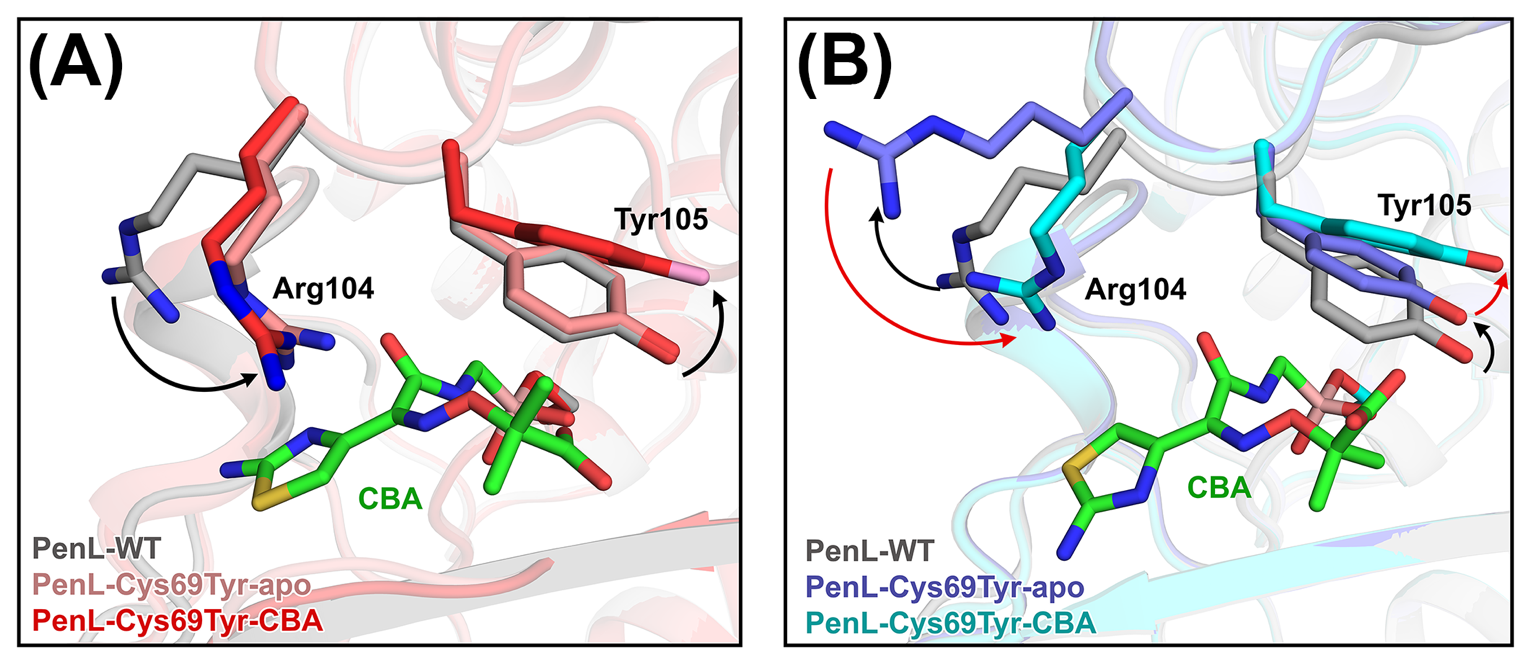
**

**Figure S7. Configurations of Arg104 and Tyr105 in lid.** PenL-WT is colored gray and superimposed on PenL-Cys69Tyr and PenL-Asn136Asp in apo- and CBA-bound forms

**Molecular dynamics (MD) simulation**

MD simulation of all three structures (PenL-WT, Cys69Tyr, and Asn136Asp) was performed in the explicit solvent environment for 50 ns duration to gain the insights into structural stability followed by the production of MD properties (Figure S8). In the initial 5 ns, The RMSD of Cα atoms of all the three structures has been gradually increased and then reaches a plateau in the following time (Figure S8A). During the 50 ns simulation, all the structures were stable but the major three catalytic regions namely Ω -loop, Lid and β3-β4 of the PenL-Cys69Tyr and PenL-Asn136Asp have robustly fluctuated compare to the PenL-WT. The high degree of Ω-loop fluctuations sterically impelled the β3-β4 strand from its initial position and the lid region oscillated in an eccentric Y-axis direction (Figure S8A and S8B). Apart from this, the radius of gyration (Rgyr) plots depicted the extended unfolding property and momentary expansion of those specific regions in the two PenL-ESBLs (Figure S8B and S8C), which strongly suggested the elevated adaptability of substrate binding and recognition towards the hydrolysis of CAZ in terms of protein dynamism.


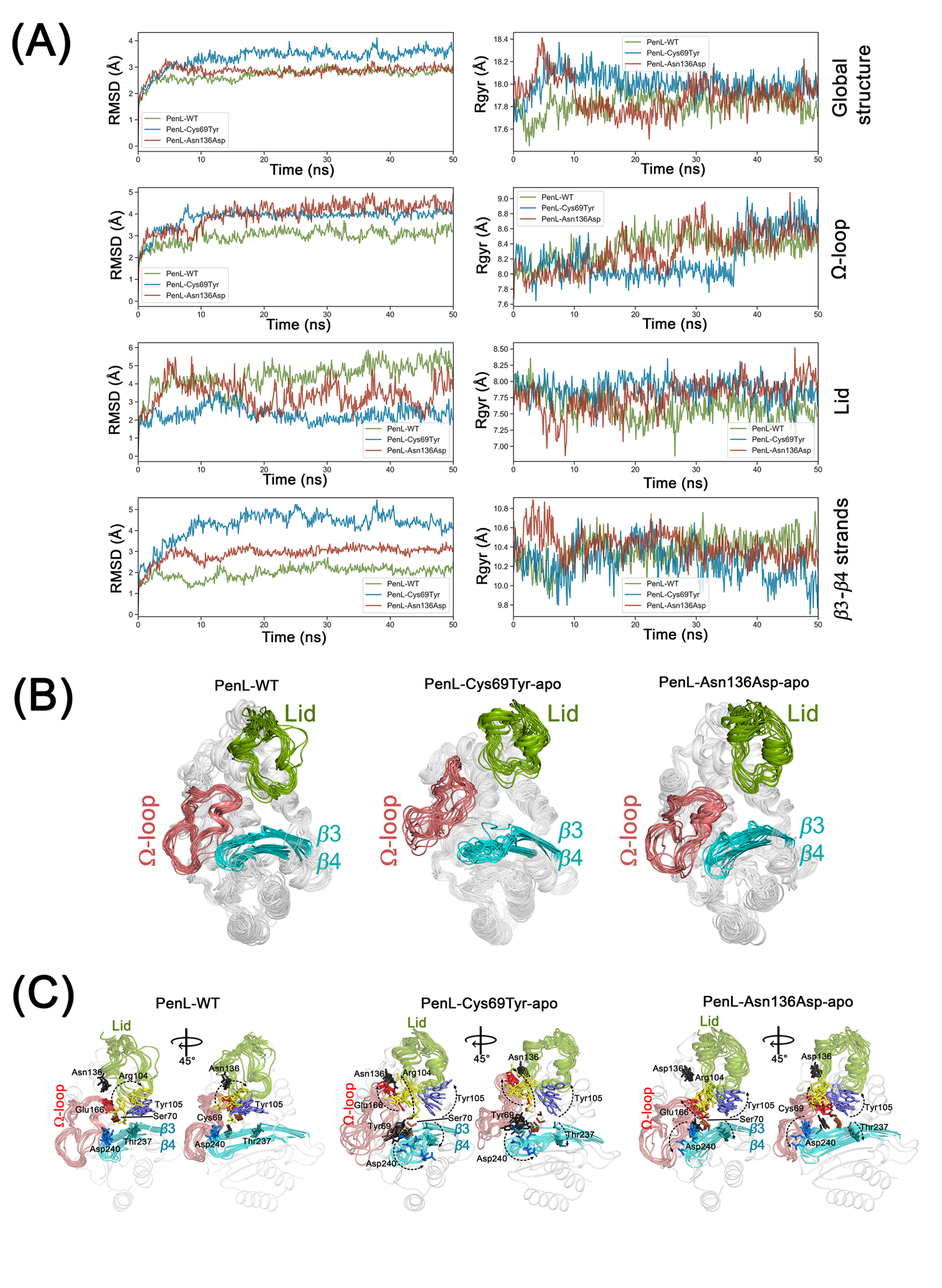


**Figure S8. Molecular dynamics analysis of PenL-NCR-ESBLs.** The simulation was run for 50 ns. (A) Root-mean-square deviation (RMSD) (left panel) and Radius of gyration (Rgyr) (right panel) of PenL-NCR-ESBLs and PenL-WT for global structure and recognition ensemble, respectively. (B) Representative ensemble of the fluctuation on the three essential segments during 50 ns. (C) Movement of representative residues involving catalysis and recognition during simulation.

**
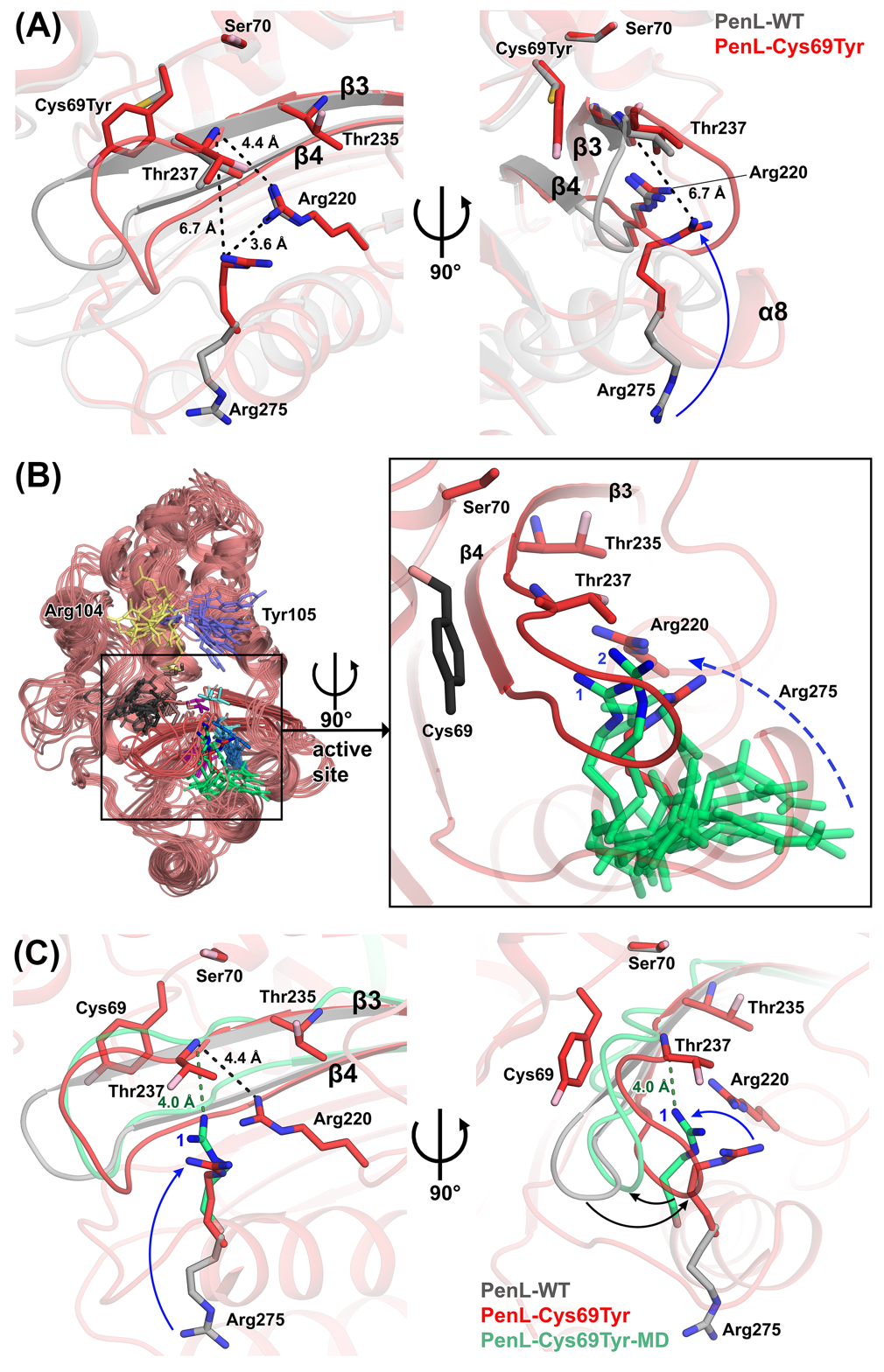
**

**Figure S9. Positive charge addition to the active site in PenL-Cys69Tyr ESBL.** Dislocation of Arg275 accompanied by dynamic fluctuation possibly enhance the positive charge distribution around the oxyanion hole in the active site. (A) Arg275 (red) in the ESBL approaches nearer to Thr237 with 6.7 Å than that of wild type (gray). (B) Representative configurations of Arg275 (green) during MD. Of the configurations, two frames (1 and 2 of left panel) position closer to the N atom of the Thr237 on β3 than that in crystal structure (red). (C) Configuration 1 of Arg275 (green) positions adjoining Thr237 in the distance of 4.0 Å. As Arg275 moves toward to the active site from WT to crystal structure, and to MD configuration, the disordered loop by the mutation infiltrate into the oxyanion hole around active site. Considering above factors, the drastic displacement of Arg275 is likely responsible for the positive charge distribution to the active site.

**Movie clips**

**Movie S1.** Comparison and movement of the critical residues for CAZ binding between PenL-WT, PenL-Cys69Tyr-apo, and PenL-Cys69Tyr-CBA.

**Movie S2.** Comparisons of electrostatic properties in the active site between PenL-WT, PenL-Cys69Tyr-apo, and PenL-Cys69Tyr-CBA.

**Movie S3.** Comparison and movement of the critical residues for CAZ binding between PenL-WT, PenL-Asn136Asp-apo, and PenL- Asn136Asp -CBA.

**Movie S4.** Molecular dynamics of PenL-Asn136Asp-apo

**Table S1. Summary of X-ray crystallographic analysis**

|  | PenL-Cys69Tyr-apo | PenL-Cys69Tyr-CBA | PenL-Asn136Asp-apo | PenL-Asn136Asp-CBA |
| --- | --- | --- | --- | --- |
| **PDB ID** | 6AFM | 6AFN | 6AFO | 6AFP |
| **Data collection** |  |  |  |  |
| Beam line | PAL-5C | PAL-5C | PAL-5C | PAL-5C |
| Wavelength (Å) | 0.97951 | 0.97954 | 0.97954 | 0.97857 |
| Resolution (Å) | 50 – 1.3 (1.32 – 1.3) | 50 – 1.4 (1.42 – 1.40) | 50 – 1.4 (1.42 – 1.40) | 50 – 1.4 (1.42 – 1.40) |
| Space group | P2_1_2_1_2_1_ | P2_1_2_1_2_1_ | P2_1_ | P2_1_ |
| Unit cell dimension |  |  |  |  |
| a, b, c (Å) | 38.48, 52.98, 122.73 | 38.66, 53.24, 122.98 | 34.99, 92.72, 68.88 | 34.88, 92.39, 68.84 |
| α, β, γ (°) | 90, 90, 90 | 90, 90, 90 | 90, 92.72, 90 | 90, 94.27, 90 |
| Total reflections | 413760 | 618880 | 618880 | 516034 |
| Unique reflections | 62299 (3048) | 50752 (2479) | 86131 (4254) | 84356 (4189) |
| R_merge_† (%) | 5.3 (9.8) | 7.8 (25.6) | 8.0 (27.1) | 8.9 (28.7) |
| Completeness (%) | 99.3 (98.4) | 99.6 (99.4) | 99.0 (100.0) | 98.6 (99.2) |
| Redundancy | 6.6 (6.5) | 12.2 (12.1) | 7.5 (7.4) | 6.1 (6.0) |
| Average I/σ (I) | 49.47 (25.74) | 43.78 (18.49) | 40.23 (10.44) | 36.43 (7.33) |
| Matthews coefficient  (Å^3^Da^-1^) | 2.18 | 2.21 | 1.95 | 1.93 |
| Solvent (%) | 43.68 | 44.33 | 37.0 | 36.45 |
|  |  |  |  |  |
| **Refinement** |  |  |  |  |
| R_work_ / R_free_ (%) | 15.00/16.77 | 14.74/16.88 | 14.86/17.35 | 14.87/17.53 |
| Protein residues | 268 | 268 | 535 | 536 |
| Waters | 484 | 371 | 755 | 629 |
| *CBA | - | 1 | - | 2 |
| RMSD |  |  |  |  |
| Bond angles (°) | 0.83 | 1.30 | 1.64 | 1.90 |
| Bond lengths (Å) | 0.005 | 0.012 | 0.015 | 0.020 |
| Average B factors (Å^2^) | 11.92 | 17.18 | 13.18 | 16.17 |
| Ramachandran plot |  |  |  |  |
| Favored (%) | 97.37 | 97.74 | 97.18 | 98.31 |
| Outliers (%) | 0.00 | 0.00 | 0.19 | 0.00 |
| Values in parentheses correspond to the shell with the highest resolution.  †R-merge=Σ*_hkl_* Σ*_i_* \|*I_i_(hkl)*-<*I(hkl)*>\|/ Σ*_hkl_* Σ*_i_* *I_i_(hkl)*, where *I_i_(hkl)* is the observed intensity and <*I(hkl)*> denotes the average intensity of symmetry-related observations.  *CBA: ceftazidime-like glycylboronic acid | | | | |

**REFERENCES**

C Chanal-Claris, D Sirot, L Bret, P Chatron, R Labia, and Sirot, J. (1997). Novel extended-spectrum TEM-type beta-lactamase from an Escherichia coli isolate resistant to ceftazidime and susceptible to cephalothin. *Antimicrob. Agents. Chemother* 41(3)**,** 715-716.

Dolinsky TJ, Nielsen JE, McCammon JA, and NA., B. (2004). PDB2PQR: an automated pipeline for the setup, execution, and analysis of Poisson-Boltzmann electrostatics calculations. *Nucleic Acids Res* 32**,** W665-W667.

Drawz, S.M., and Bonomo, R.A. (2010). Three Decades of β-Lactamase Inhibitors. *Clin. Microbiol. Rev.* 23(1)**,** 160-201. doi: 10.1128/CMR.00037-09.

George Minasov, Xiaojun Wang, and Shoichet, B.K. (2002). An ultrahigh resolution structure of TEM-1 beta-lactamase suggests a role for Glu166 as the general base in acylation. *J. Am. Chem. Soc.* 124(19)**,** 5333–5340.

J P Quinn, D Miyashiro, D Sahm, R Flamm, and Bush, K. (1989). Novel plasmid-mediated beta-lactamase (TEM-10) conferring selective resistance to ceftazidime and aztreonam in clinical isolates of Klebsiella pneumoniae. *Antimicrob. Agents. Chemother* 33(9)**,** 1451–1456.

Jurrus E, Engel D, Star K, Monson K, Brandi J, Felberg LE, et al. (2018). Improvements to the APBS biomolecular solvation software suite. *Protein Science* 27**,** 112-128.

M. Cecilia Orencia, Jun S. Yoon, Jon E. Ness, Stemmer, W.P.C., and Stevens, R.C. (2001). Predicting the emergence of antibiotic resistance by directed evolution and structural analysis. *Nat Struct Biol.* 8**,** pages 238–242.

Olsson, M.H., Chresten R. Søndergaard, Michal Rostkowski, and Jensen, J.H. (2011). PROPKA3: Consistent Treatment of Internal and Surface Residues in Empirical pKa Predictions. *J. Chem. Theory Comput.* 7(2)**,** 525-537.

Palzkill, T. (2018). Structural and Mechanistic Basis for Extended-Spectrum Drug-Resistance Mutations in Altering the Specificity of TEM, CTX-M, and KPC β-lactamases. *Front Mol Biosci* 5(16).

Papp-Wallace, K.M., Becka, S.A., Taracila, M.A., Winkler, M.L., Gatta, J.A., Rholl, D.A., et al. (2016). Exposing a β-Lactamase “Twist”: the Mechanistic Basis for the High Level of Ceftazidime Resistance in the C69F Variant of the Burkholderia pseudomallei PenI β-Lactamase. *Antimicrobial Agents and Chemotherapy* 60(2)**,** 777-788. doi: 10.1128/aac.02073-15.

Papp-Wallace, K.M., Taracila, M.A., Gatta, J.A., Ohuchi, N., Bonomo, R.A., and Nukaga, M. (2013). Insights into β-Lactamases from Burkholderia Species, Two Phylogenetically Related yet Distinct Resistance Determinants. *J. Biol. Chem.* 288(26)**,** 19090-19102.

Rigos, C.F., Santos, H.d.L., Jr, G.T., Ward, R.J., and Ciancaglini, P. (2006). Influence of enzyme conformational changes on catalytic activity investigated by circular dichroism spectroscopy. *Biochem Mol Biol Educ.* 31(5)**,** 329-332.

Yi, H., Choi, J.M., Hwang, J., Prati, F., Cao, T.-P., Lee, S.H., et al. (2016). High adaptability of the omega loop underlies the substrate-spectrum-extension evolution of a class A β-lactamase, PenL. *Scientific Reports* 6(1). doi: 10.1038/srep36527.
